# *In Silico* Structural Evaluation of Short Cationic Antimicrobial Peptides

**DOI:** 10.3390/pharmaceutics10030072

**Published:** 2018-06-21

**Authors:** Ilaria Passarini, Sharon Rossiter, John Malkinson, Mire Zloh

**Affiliations:** 1 School of Life and Medical Sciences, University of Hertfordshire, College Lane, Hatfield AL10 9AB, UK; i.passarini@herts.ac.uk (I.P.); s.rossiter@herts.ac.uk (S.R.); 2 UCL School of Pharmacy, University College London, 29/39 Brunswick Square, London WC1N 1AX, UK; j.malkinson@ucl.ac.uk; 3 Faculty of Pharmacy, University Business Academy, Trg mladenaca 5, 21000 Novi Sad, Serbia; 4 NanoPuzzle Medicines Design, Business & Technology Centre, Bessemer Drive, Stevenage SG1 2DX, UK

**Keywords:** cationic antimicrobial peptides (AMPs), amphipathic conformation, molecular dynamics, protein structure prediction, dodecylphosphocholine (DPC) micelles

## Abstract

Cationic peptides with antimicrobial properties are ubiquitous in nature and have been studied for many years in an attempt to design novel antibiotics. However, very few molecules are used in the clinic so far, sometimes due to their complexity but, mostly, as a consequence of the unfavorable pharmacokinetic profile associated with peptides. The aim of this work is to investigate cationic peptides in order to identify common structural features which could be useful for the design of small peptides or peptido-mimetics with improved drug-like properties and activity against Gram negative bacteria. Two sets of cationic peptides (AMPs) with known antimicrobial activity have been investigated. The first reference set comprised molecules with experimentally-known conformations available in the protein databank (PDB), and the second one was composed of short peptides active against Gram negative bacteria but with no significant structural information available. The predicted structures of the peptides from the first set were in excellent agreement with those experimentally-observed, which allowed analysis of the structural features of the second group using computationally-derived conformations. The peptide conformations, either experimentally available or predicted, were clustered in an “all vs. all” fashion and the most populated clusters were then analyzed. It was confirmed that these peptides tend to assume an amphipathic conformation regardless of the environment. It was also observed that positively-charged amino acid residues can often be found next to aromatic residues. Finally, a protocol was evaluated for the investigation of the behavior of short cationic peptides in the presence of a membrane-like environment such as dodecylphosphocholine (DPC) micelles. The results presented herein introduce a promising approach to inform the design of novel short peptides with a potential antimicrobial activity.

## 1. Introduction

The surge of multidrug resistant microorganisms and the lack of new antibiotics present a major challenge to modern medicine [1]. Naturally-occurring cationic peptides often possess antimicrobial properties [2] and represent a promising class of lead compounds to be selected for the design of new antibiotics. However, of the nearly 5000 cationic antimicrobial peptides (AMPs) described to date, fewer than 100 are currently undergoing clinical trials [3], possibly due to the challenges related to the development of protein-based drugs. Several resources are available for predicting the 3D conformations of peptides, such as the online software PEP-FOLD [4], as well as their antimicrobial potential [5]. Three pathways are usually followed in efforts to obtain novel peptides: modification of existing templates through bioinformatics methods, often looking at the modification of the primary sequences; biophysical modelling, such as molecular dynamics simulations, which can also take into account the effect of the environment on the conformations; and screening of libraries which may focus on the structure activity relationship (SAR) [6,7]. However, the complexity of problems associated with *in vivo* application of these molecules and understanding their mechanism of action calls for a continuous improvement of these methods, with the aim to obtain antimicrobial agents with improved pharmacological profiles.

Despite their mechanism(s) of action still not being fully understood, it is broadly accepted that AMP play a role in the alteration of the bacterial membrane and wall systems, with the consequent disruption of the proton motive force and the general viability of the organism [8]. Although the experimental studies, such as NMR spectroscopy, can provide information on the structures and orientation of peptides bound to micelles, *in silico* studies such as molecular dynamics (MD) are often utilized for the study of the interaction of AMPs with membrane-like structures [9]. Such approaches are important for the design of the novel peptides as the hypothesis can be tested before time-consuming and resource-demanding synthesis and purification.

Therefore, the aim of our work was to investigate both the sequences and conformations of AMPs to possibly identify common features that can inform the design of novel short peptides or small peptide mimetics with antimicrobial potential and a more favorable pharmacokinetic profile. To this aim, a library of active peptides with known 3D structures was initially created and clustered and the centroids analyzed. This confirmed the experimental evidence that the majority of these molecules often contain basic amino acid residues in close proximity to aromatic residues and that they assume an amphipathic conformation [2,5]. Furthermore, it was shown that their predicted conformations are in excellent agreement with those experimentally observed in solution. The structures of the second set of AMPs, with no experimental data about their conformation, were predicted *in silico* before being clustered. The judicious analysis of the centroids of the most populated clusters suggested that these peptides with activity against Gram negative bacteria also assume amphipathic conformations, although no particular predominance of basic or aromatic amino acid residues was apparent. Finally, it was demonstrated that the interactions between short AMPs and a membrane-like environment can be investigated *in silico* using the predicted structure as a starting point. The results of this work provide important insights in structural features of AMPs with activity against Gram negative bacteria and indicate that a combination of *in silico* approaches can aid efforts to design novel AMPs.

## 2. Materials and Methods

### 2.1. Data Collation

The first dataset of peptides with known 3D structures was created using a search for a string “antimicrobial peptides” in the protein databank (PDB) [10]. The search results were filtered to select structures derived from solution-state NMR. All cyclic peptides, dimers or multimers and peptide-receptor complexes were not considered and no other specific restrictions, e.g., size, charge, origin or mechanism of action, were included in the search. All peptides were active either against Gram positive and/or negative strains. A library of 117 PDB entries was thus created based on results retrieved at the time of the search.

All short peptides were shortlisted from this first set, namely those under 15 residues in length. This resulted in a list of 12 molecules, which were then further divided into two groups, containing structures determined either in the presence (Table 1) or absence (Table 2) of micelles.

A second set of short cationic antimicrobial peptides was created through a literature search and cross-referenced against existing on-line databases, such as the “Antimicrobial Peptide Database” [23]. They were required to be active against Gram negative strains with minimum inhibitory concentrations (MICs) below 128 μg/mL, have a primary sequence ranging between 9 and 15 amino acids and carry at least one net positive charge at neutral pH. As opposed to the first set of peptides, no information about their conformations was available in the literature at the time of the data collation.

### 2.2. Structure Prediction and Molecular Dynamics Simulation

The 3D conformations of all short peptides from the first set (containing 15 amino acid residues or fewer) were initially predicted using the online software PEP-FOLD [4]. Due to software limitations, the conformation can only be predicted for peptides with a primary sequence between 9 and 36 residues. The primary sequence is submitted using a single letter code and the program predicts the 3D conformation by assembling predicted conformations of short local sequences using a greedy procedure driven by a coarse-grained energy score. The results were then imported into Maestro as .pdb files and the *C*-terminus was amidated when appropriate.

The initial predictions were then submitted to molecular dynamics (MD) simulations using Desmond [24] and the OPLS 2005 all atoms force field [25] (Maestro version 11.0.014, Schrödinger LLC, New York, NY, USA). All peptides were prepared for the simulation using Protein Preparation wizard by adding all hydrogen atoms and setting the protonation states of all ionizable groups for pH 7. Each peptide was fully solvated using an explicit solvent (SPC water model) with the box size 10 Å larger than the size of the molecule in all directions using System Builder. Ions were added to mimic physiological conditions with a 0.15 M concentration of NaCl, and including Cl^-^ as counter ions to neutralize the system. Each system was minimized until the norm of the energy gradient was <0.1 kcal/mol. Furthermore, the whole system was simulated for 10 ns at 300 K under constant pressure and temperature (NPT) conditions. The results of the simulations were saved as trajectories of structures at every 5 ps. Trajectories and structures extracted from the final frame were used for HC and analysis. Conformations at 0, 1.2 and 10 ns were superimposed on the experimental PDB structures using the α-carbon alignment tool featured in Maestro.

The aforementioned method was also applied for the 3D structures prediction of peptides from the second set, namely by subjecting the PEP-FOLD predicted conformations to 1.2 ns MD simulations under the same conditions reported earlier.

### 2.3. Hierarchical Clustering

Hierarchical clustering (HC) was conducted on the two different sets of peptides, with experimentally-determined and predicted structures. A list of the 3D structures in pdb format was generated as a text file and submitted to the MaxCluster software for clustering [26]. HC with average linkage based on root mean square deviation (RMSD) was performed, with a threshold set at 700 for the first set and 800 for the second. The centroids of the most populated clusters were analyzed, looking for common features in the amino acid sequences and in the tertiary structures. The surfaces of all peptides were also generated using the structure analysis tool featured in Maestro (version 11.0.014, Schrödinger LLC, New York, NY, USA) to evaluate and compare surface hydrophobic and hydrophilic properties.

### 2.4. Molecular Dynamics Simulation of Peptides in the Presence of Micelles

Finally, the short antimicrobial peptide Anoplin was selected as a case study for the investigation of the behavior of short AMPs in presence of a membrane-like environment [27]. The NMR structure of Anoplin combined with dodecylphosphocholine (DPC) micelle is available as PDB entry 2MJT [11]. In order to test the suitability of this system and of the software used, 2MJT PDB file was initially downloaded (including the micelle). A fully solvated system was prepared with 10 Å water buffer, 0.15 M NaCl, and Na^+^ and Cl^−^ as counter ions. A 10 ns MD simulation was conducted using Desmond and OPLS2005 force field (Maestro version 11.0.014, Schrödinger LLC, New York, NY, USA). This was followed by a set of MD simulations on a system built to recreate the experimental conditions described by Uggerhoj [11]. Therefore, a micelle formed by 65 DPC molecules was initially built using Packmol [28]. The terminal carbon atoms of the hydrophobic chains were constrained in the center of the box around a sphere of 4 Å radius, so that the hydrophobic tails would point towards the centre of the micelle. Conversely, all nitrogen atoms belonging to the choline moiety were constrained around a sphere of radius 18 Å, so that the polar heads of 20 Å long DPC molecules would point towards the external aqueous environment. A 10-mM phosphate buffer was also included, with three diphosphate and one monophosphate molecules randomly distributed in the system. Finally, a structure of Anoplin was also included at a random position 10 Å away from the micelle. Two different systems were built with Anoplin (1) in an extended conformation and (2) in a conformation predicted using PEP-FOLD. A water buffer of 80 Å sides was built around the systems using System Builder in Desmond, including 15 mM NaCl and relevant counter ions. 10 ns simulations were then run using Desmond with the OPLS2005 force field [25], keeping constant atmospheric pressure (1.01325 bar) and room temperature (300 K).

## 3. Results

### 3.1. Investigation of a Set of Antimicrobial Peptides with Known Confomation

A set of 117 PDB entries corresponding to NMR-derived conformations of antimicrobial peptides was created (Table S1). These entries were clustered according to the lowest possible root mean square difference (RMSD) in an “all vs. all” fashion (Table 3). The four most populated clusters with their centroids were analyzed in detail, while the structures of the remaining clusters’ centroids are shown in Figure S1.

The 3D conformations of all peptides containing 15 residues or fewer were then predicted, by submitting the conformations obtained through the online software PEP-FOLD to 10 ns MD simulations. The backbones of the conformations simulated at 10, 1.2 and 0 ns (the latter corresponding to the PEP-FOLD prediction) were superimposed to the corresponding PDB entries, which were obtained from NMR experiments. The structures were compared using RMSD (Table 4).

### 3.2. Investigation of a Set of Antimicrobial Peptides with No Known Conformation

A search for short cationic antimicrobial peptides active against Gram negative strains was conducted, providing a set of 63 molecules with no experimental available data on their 3D structures (Table S2). Their initial conformations were predicted using PEP-FOLD, and they were used to build the systems for 1.2 ns MD simulations. The structures extracted from the final frames of corresponding trajectories were clustered in an “all vs. all” fashion by calculating the lowest possible RMSD. This provided 13 clusters, one of which contained 25 peptides and one 20 (Table 5). All other clusters were only formed by one to three peptides, and the corresponding centroids are shown in Figure S10.

### 3.3. A System to Study the Interaction of Short Antimicrobial Peptides with a Membrane-Like Structure

A system to study the behaviour of short cationic peptides in presence of a membrane-like environment, such as dodecylphosphocholine (DPC) micelles, was evaluated. The PDB entry 2MJT was selected as the template, as it corresponds to the NMR-derived conformation of the antimicrobial peptide Anoplin in the presence of DPC micelles. Firstly, 10 ns MD simulation on the system was performed in aqueous buffer to test the suitability of the software. The conditions of the NMR experiment were then recreated *in silico*. An MD simulation of a system that included the extended structure of Anoplin did not result in a folded peptide as expected, as the simulation time of 1.2 ns was short. In fact, the extended peptide would not provide an adequate interface enabling the formation of favourable interactions with the micelle. However, when the PEP-FOLD predicted conformation of the peptide was included in the system, the molecule could be seen embedding into the micelle whilst assuming the typical amphipathic disposition of the side chains.

## 4. Discussion

### 4.1. Investigation of a Set of Antimicrobial Peptides with Known Confomations

A library of 117 antimicrobial peptides (AMPs) with experimentally-known 3D structures was initially created (Table S1). The corresponding PDB entries were hierarchically clustered through the MaxCluster software [26]. The distance between two items was calculated on the root mean square deviation (RMSD), using the Kabsch rotation matrix [29,30]. The software calculates the RMSD by finding the superposition between two items which gives the lowest score. Average linkage clustering was selected over single and maximum linkage as it could be less influenced by outliers. In fact, single linkage is based on the shortest distance between any two members; maximum linkage is based instead on the largest distance between any two items; whilst average linkage adopts the average of all pairwise distances between members of the two groups which are being compared. A threshold was set at 700, aiming to obtain an acceptable number of clusters of peptides with similar folds. These provided 14 clusters, which are reported in Table 3. The centroids of the four most populated clusters were analysed in depth.

**Figure 1:**
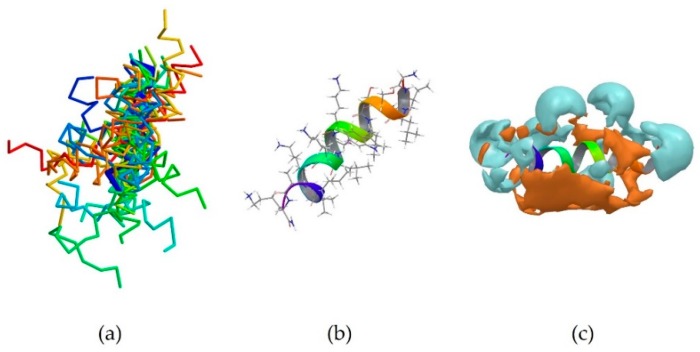
(**a**) Superimposition of all members of cluster 1 onto the backbone of the centroid 5MMK shown in thick blue trace representation. 19 members out of 24 in this cluster assume at least a partial α-helix conformation (thin, coloured α-carbon traces); (**b**) Stick representation of the experimentally-derived conformation of 5MMK. The ribbon shows how the antimicrobial peptide 5MMK assumes an α-helix conformation; (**c**). Side view of the PDB entry 5MMK, highlighting the polar regions of the surface. The hydrophilic part is shown in cyan, whilst the hydrophobic component is displayed in amber.

5MMK is the centroid of cluster 1, which is the most populated one, containing 24 peptides. It corresponds to the antimicrobial peptide HYL-20, whose sequence is GILSSLWKKLKKIIAK-NH_2_ [31]. A predominance of the basic amino acid lysine can be observed. It should be noted that Lys8 in particular is positioned next to the aromatic amino acid Trp7. 5MMK assumes an α-helix secondary structure (Figure 1b). It was observed that 19 members out of 24 in this cluster also assume at least a partial α-helix conformation (Figure 1a). Hydrophobic and hydrophilic regions of the 5MMK surfaces were calculated with Maestro (version 11.0.014, Schrödinger LLC, New York, NY, USA) and it can be observed that the hydrophilic and hydrophobic side chains are clustered on the two opposite sides of the protein, with a predominance of the hydrophilic portion, which can be attributed to the positively-charged amino acids (Figure 1c). 5MMK therefore assumes an amphipathic configuration, as expected [32].

**Figure 2:**
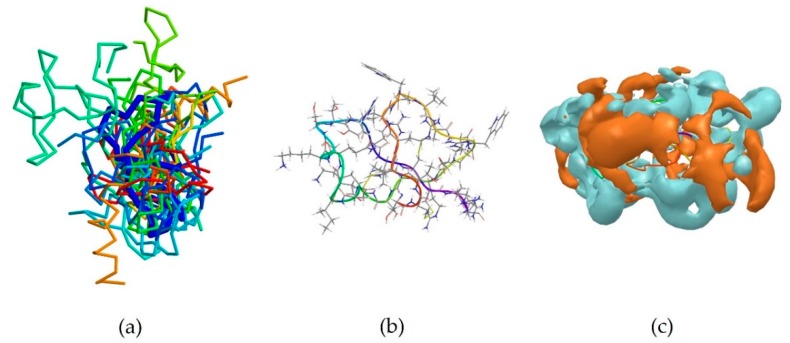
(**a**) Superimposition of all members of cluster 2 onto the backbone of the centroid 2MXQ (thick blue trace). The majority of the members of this cluster assume a partial β-sheet configuration, with only 2K35, 2MMM, 2MN5 and 2N8D including α-helix regions; (**b**) Stick and ribbon representations of the experimentally-derived conformation of 2MXQ which does not assume a definite conformation; (**c**) PDB structure 2MXQ, corresponding the defensin-like peptide DEFA1 is here shown. The hydrophilic region of the molecule surface is coloured in cyan, whilst the hydrophobic portion is shown in amber.

2MXQ was also investigated, being the centroid of the second most populated cluster, which includes 14 peptides (Table 3). It corresponds to DEFA1, a 34-amino-acid peptide, whose sequence is SCTCRRAWICRWGERHSGKCID9KGSTYRLCCRR [33]. DEFA1 has a rather longer sequence if compared to the first centroid analysed. However, a predominance of basic amino acid residues, in this case arginine, can still be observed, which in several cases are close to either another arginine (Arg5-Arg6, and Arg33-Arg34), or next to an aromatic amino acid, such as tryptophan, histidine or tyrosine (Arg11-Trp12, Arg15-Trp16, Tyr28-Arg29). In contrast with HYL-20, it does not assume an α-helix nor a definite conformation in solution, possibly suggesting a flexible peptide (Figure 2b) [33]. Despite this, the majority of the members belonging to cluster 2 (11 out of 14) adopt a β-sheet conformation in a part of their sequence, in clear contrast with cluster 1 previously investigated (Figure 2a). The investigation of the polarity of the surface also showed no clear predominance of one region over the other. However, a separation between the two can still be seen, with the hydrophobic portion appearing “sandwiched” between two hydrophilic areas, suggesting an amphipathic nature of the peptide (Figure 2c).

**Figure 3:**
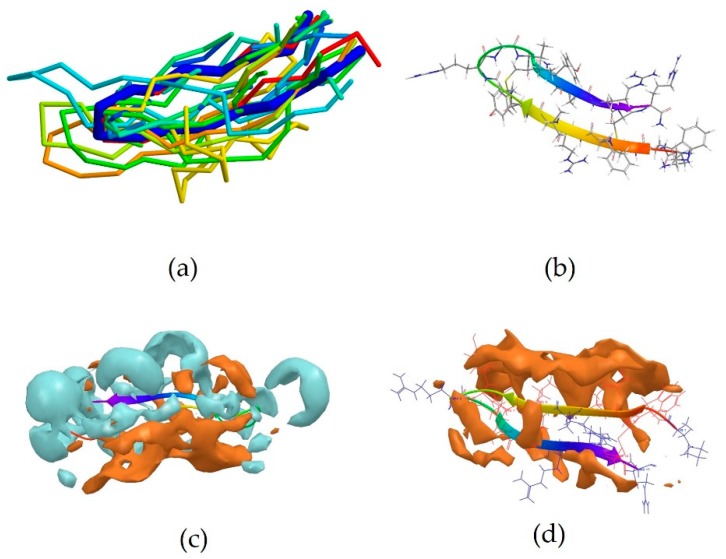
(**a**) Peptides populating cluster 3 superimposed onto the backbone of the centroid 2RTV (thick blue trace). No member of this cohort presented an α-helix arrangement; (**b**) Experimentally-derived 3D conformation of the centroid 2RTV; (**c**) The hydrophobic (amber) and hydrophilic (blue) regions on the surface of 2RTV are shown. It can be observed that Tachyplesin I displays a predominantly hydrophilic nature; (**d**) Detail of the hydrophobic region.

2RTV is the centroid of cluster 3, which contains 13 peptides (Table 3). It corresponds to the NMR-derived structure of the peptide Tachyplesin I in water [34]. It is a *C*-terminally amidated 18-residue peptide, whose sequence is KWCFRVCYRGICYRRCR-NH_2_. It can be observed that it is also rich in basic amino acids, which are responsible for the overall positive charge of the peptide at neutral pH, with 5 arginine and one lysine residues. It can also be noted that these residues are always close to an aromatic residues, such as tryptophan, tyrosine or phenylalanine, with the exception of Arg15, which is however next to another Arg residue, and the terminal Arg18. 2RTV forms two β-sheets connected by a loop (Tyr8, Arg9, Gly10, Ile11) and linked together by two disulfide bridges (Cys16-Cys3 and Cys12-Cys17) (Figure 3b). The majority of the members of this cluster also assume a β-sheet conformation (Figure 3a). Although less evident than for 5MMK, here again two distinct regions can be identified: a hydrophilic portion is formed by the *N*-terminal Lys and *C*-terminal Arg and at the loop around Arg9 (Figure 3c), whilst a hydrophobic section is found around the β-sheet between Tyr8 and Trp2 (Figure 3d).

**Figure 4:**
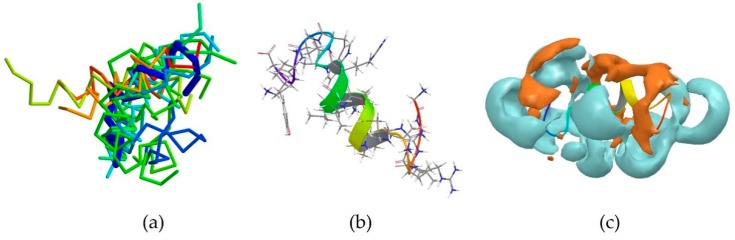
(**a**) Members of cluster 4 are shown superimposed to the centroid 2NDC (thick blue trace). 9 members out of 13 also assume at least a partial α-helical conformation; (**b**) Experimentally-derived conformation of peptide 2NDC; (**c**) PDB entry 2NDC with its surface investigated. Hydrophobic areas are shown in amber, hydrophilic in cyan.

The peptide 2NDC is the centroid of cluster 4 which also contains 13 peptides (Table 3). Its sequence is GGLRSLGRKILRAWKKYG, which again is characterized by the presence of basic Arg and Lys residues [3]. In particular, Lys15 can be found next to aromatic Trp14 and Lys16 is next to aromatic Tyr17. It assumes a partially α-helical secondary structure, like the majority of the members of this cluster (Figure 4a,b). Unsurprisingly, its surface is also largely hydrophilic (Figure 4c). This peptide is helical in the middle of the sequence.

Most of the NMR-derived conformations of centroids of the remaining clusters feature secondary structures observed in the first four clusters, i.e., α-helixes (Figure S1b,d,h) and β-sheets (Figure S1c,f,i), while the rest adopt less ordered structures. Despite having distinct features present in the centroids of clusters 1 to 4, the members of remaining clusters do not group with those clusters due to either the presence of disulfide bonds (i.e., PDB entry 2MUH) or their larger size, which may lead to difficulty in ab initio prediction of their structure, as it is less reliable than the prediction of the shorter sequences [35].

### 4.2. Evaluation of In Silico Appraoches to Predict Conformation of Short Cationic Peptides

All peptides with known structure and with sequence length under 15 residues were shortlisted for further analysis. Their conformations were initially predicted with the online software PEP-FOLD and then submitted to 10 ns MD simulation in an aqueous buffer. The PEP-FOLD predictions (corresponding to 0 ns conformation), as well as the conformations at 1.2 and 10 ns of the simulation were superimposed onto the experimental PDB entry in order to investigate the quality of such predictions and stability of predicted structures. It can be observed that the PEP-FOLD conformations, which correspond to the starting point of the MD simulation, are generally in good agreement with experimentally-determined structures extracted from PDB entries as suggested by RMSD values (Table 4).

**Figure 5:**
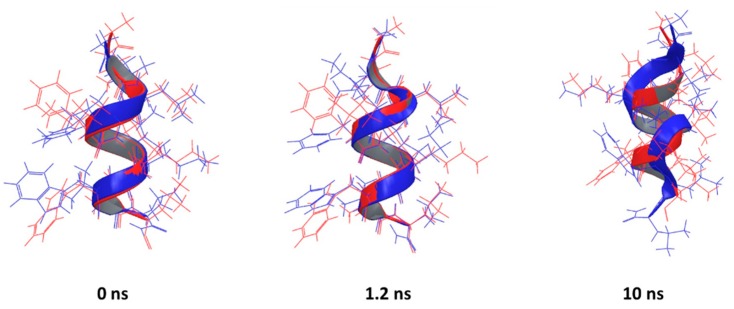
The backbone superposition of PDB entry 2MJT (red) and conformations assumed at 0, 1.2 and 10 ns of MD simulation (blue).

The best agreement between predicted and experimental conformations was observed for the peptide corresponding to the PDB entry 2MJT, with the best superposition given by the PEP-FOLD prediction (RMSD = 0.4461 Å), which is in line with the previously observed performance of PEP-FOLD software [36]. It is not only that the backbones are aligned, but most of the side-chains too. (Figure 5, 0 ns). A similar performance was observed for other helical AMPs (2F3A, 1D7N, 2JMY, 2MAA, 1T51, 2L24, 2N9A and 2NAL), with the helical parts of the backbone aligned while the termini show flexibility (Figures S2–S9).

It is important to note that PEP-FOLD predicts the conformation of peptides in an aqueous environment. The experimental structures of 2MJT, 1G89, 2F3A, 1D7N, 2JQ2, 2JMY, 2MAA, 1D6X and 1T51 were determined in the presence of micelles, believed to induce the formation of an amphipathic surface. To establish if the predicted structures are stable in water without micelles, we have conducted MD simulations of all peptides under physiological conditions. Although the RMSD decreases over simulation time in most cases, possibly indicating a flexible peptide, most of the abovementioned peptides preserve secondary structure (Figure 5 and Figures S2–S9). This suggests that the predicted structures are stable for at least 1.2 ns of simulation despite the absence of the membrane. The PEP-FOLD predicted conformations do not fully unfold in an aqueous environment and the secondary structure is preserved during the simulation in a fully solvated system but without a membrane environment. Therefore, a short simulation of 1.2 ns without the membrane could be used to explore the dynamic nature of AMPs (Table 4).

**Figure 6:**
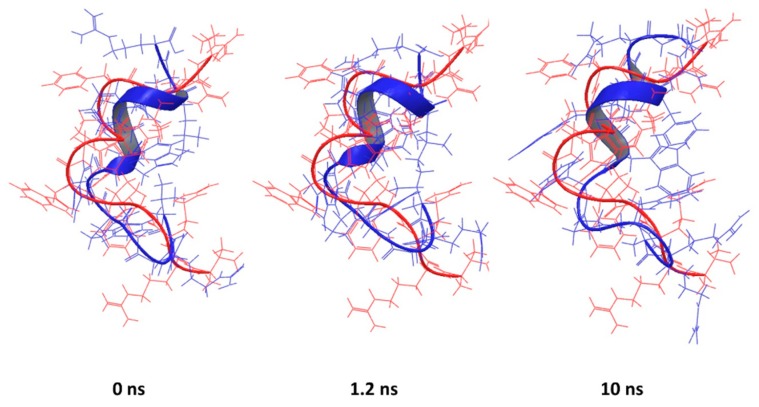
Backbone superposition of PDB entry 1D6X (red) and simulated conformations at 0, 1.2 and 10 ns (blue). No significant change was observed over the simulation time, with a slight improvement at 1.2 ns.

This is particularly true for the peptide corresponding to the PDB entry 1D6X, which had 19 NMR-based conformations stored in the downloaded file. The RMSD between the lowest energy structure and the rest of the set was in the range from 1.4 to 2.25 Å, suggesting that the peptide itself is flexible in presence of micelles. Despite that flexibility, the predicted structure has key features similar to the experimental structure that are mainly preserved during simulation (Figure 6).

**Figure 7:**
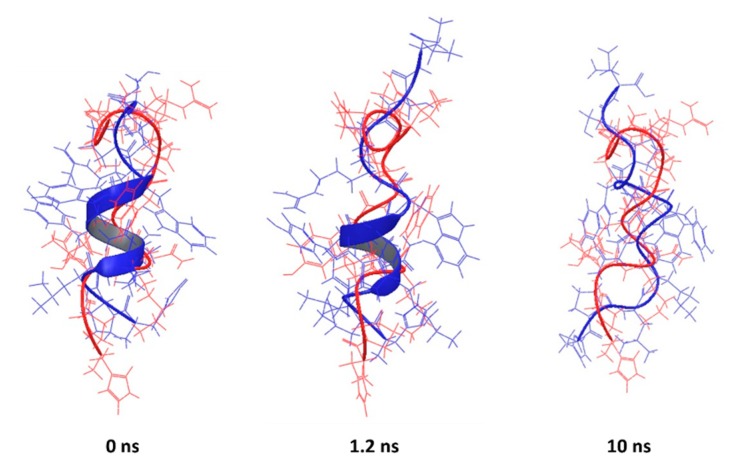
Backbone superposition of PDB entry 2JQ2 (red) and simulated conformations at 0, 1.2 and 10 ns (blue). NMR experiments show that 2JQ2 assumes relaxed coiled conformations, whilst the PEP-FOLD prediction provides a partially α-helical structure, which progressively deteriorates over the simulation time.

Similar behaviour is observed for the peptide corresponding to the PDB entry 2JQ2, for which the α-helix content was overestimated (Figure 7), as well as PDB entry 2MAA (Figure S5). It was observed that the simulation relaxed the structure into a conformation with a higher RMSD, but some of the key features became similar to the experimentally-determined conformation.

**Figure 8:**
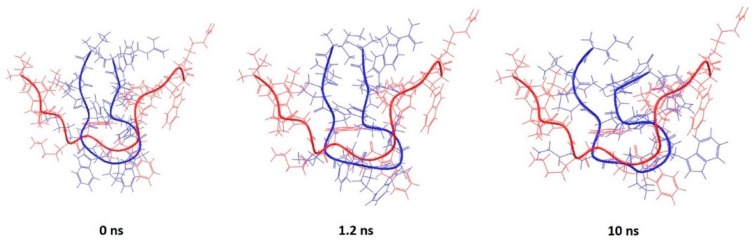
Backbone superposition of PDB entry 1G89 (red) and conformations at 0, 1.2 and 10 ns of MD simulation (blue).

There was only one peptide in the set of the short peptides that did not have helical structure, which was extracted from the PDB entry 1G89. It assumes a “U” shape with rather flexible ends (a range of RMSD for the experimentally-determined structures was from 0.6 to 1.9 Å). The absence of the micelle and absence of intramolecular interactions due to the intrinsic nature of the peptide led to a much higher RMSD between the predicted and experimental structures (RMSD = 6.4 Å). However, it can be observed that the central part of the peptide sequence (hydrophobic and possibly responsible for driving bioactive conformation) overlaps better than the termini (Figure 8).

These results indicate that, despite the absence of a membrane-like environment, PEP-FOLD complemented with molecular dynamics simulation of 1.2 ns can be used for predicting AMP structure and to explore the dynamics nature of these peptides, especially those that have propensity for forming an α helix.

### 4.3. Common Structural Features of Antimicrobial Peptides with Activity against Gram Negative Bacteria

Based on these observations, the 3D structures of 63 short cationic antimicrobial peptides with no known 3D structure were predicted by submitting the PEP-FOLD-obtained conformations to 1.2 ns MD simulation. It was chosen to only consider short peptides in anticipation of an improved pharmacokinetic profile and driving drug like properties during the design of novel molecules. The resulting conformations were once again clustered using the MaxCluster command and the resulting clusters were analysed for common features [26].

**Figure 9:**
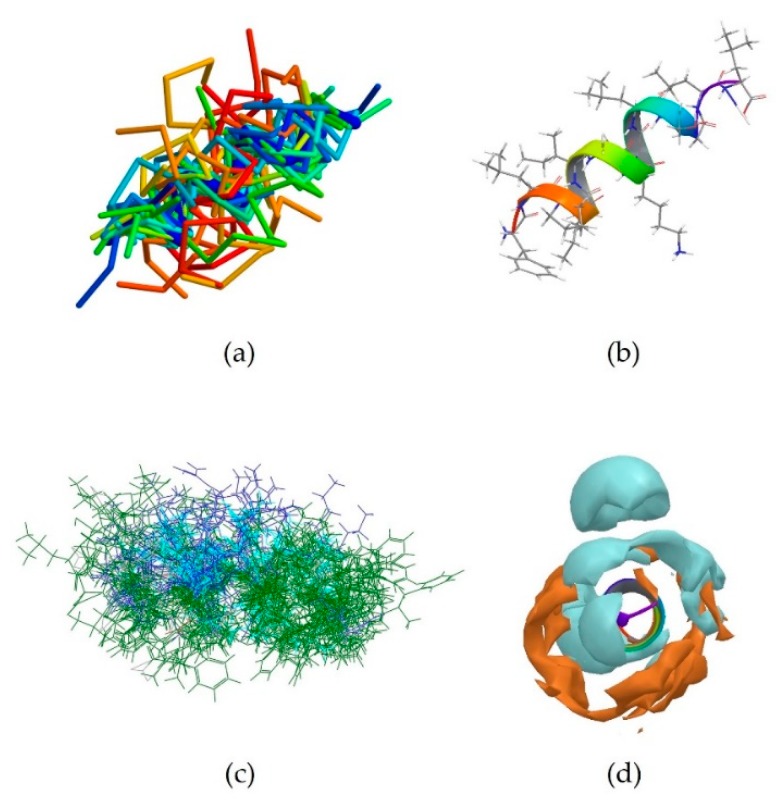
(**a**) Backbone superposition of all members of cluster 1 onto the centroid VCP-VT1 (thick blue trace); (**b**) Predicted conformation of VCP-VT1 after 1.2 ns MD simulation; (**c**) The peptides belonging to cluster 6 are shown superimposed and the residues are coloured according to their electrical properties: hydrophobic residues in green, positively-charged in blue, polar uncharged in cyan. A few peptides also contain negatively-charged residues (red), however the overall charge of the molecule is still positive. Two distinct areas can be observed, with a positively-charged portion and a predominant hydrophobic or uncharged region; (**d**) The surface of the centroid CVP-VT1 is shown along the *N*-terminal side, with hydrophilic areas in cyan and hydrophilic regions in amber. A clear distinction can be observed between the two.

Peptide VCP-VT1 is the centroid of the most populated cluster, which contains 25 peptides (Table 5). Its sequence is FLPIIGKLLSGLL. It only contains one aromatic amino acid residue (Phe1), and one basic residue (Lys7) and it maintains an α-helical conformation after 1.2 ns MD simulation, like the majority of the members of this cluster (Figure 9a,b). All peptides in cluster 1 were also superimposed along the α-carbon atoms using the superposition tool featured in Maestro. The residues were colored according to their properties. A predominance of hydrophobic and electrostatically positively-charged residues was observed. Interestingly, a clear separation in the disposition of the two sets of residues was noted, with all the positively-charged side chains converging towards one side of the peptide (Figure 9c). This observation complies with the hypothesis that these peptides assume an amphipathic 3D conformation [2], with a charged region responsible for binding to the negatively-charged LPS on the surface of Gram negative bacteria, and a predominantly hydrophobic nature allowing the peptide to then penetrate the membrane. The surface of the centroid VCP-VT1 was also analysed in terms of polarity, and a clear distinction between hydrophilic and hydrophobic areas could be observed (Figure 9d), in accordance with what was discussed for the peptides with experimentally-known conformations.

**Figure 10:**
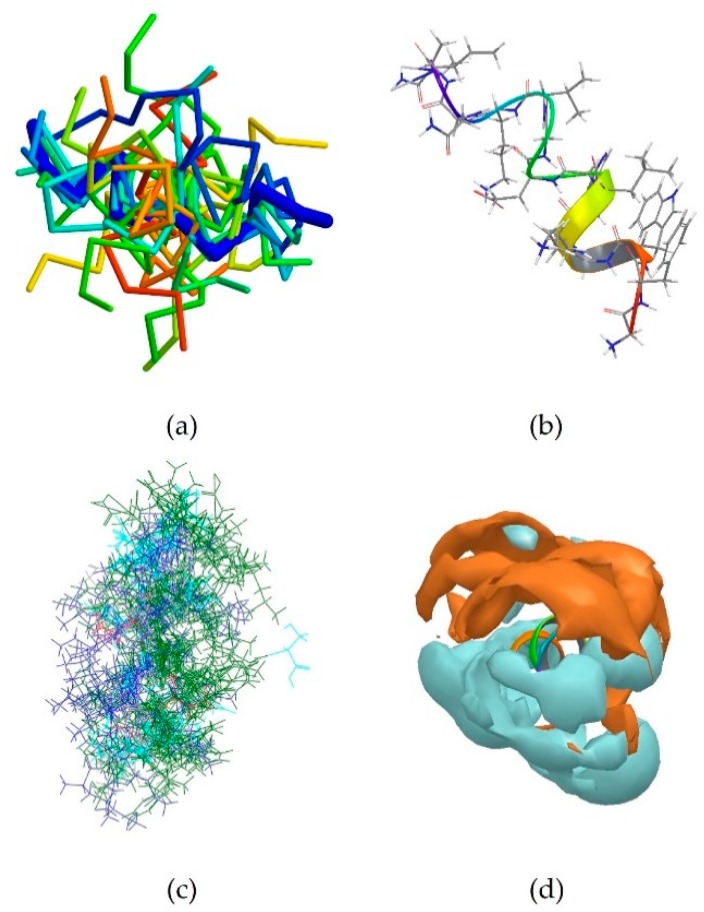
(**a**) The members of cluster 2 are shown superimposed to the centroid UyCT1 (thick blue trace); (**b**) Predicted conformation of UyCT1; (**c**) Side view of the peptides forming cluster 2 superimposed along the α-carbon. A colour scheme was applied according to residue properties: hydrophobic residues in green, positively-charged in blue, polar uncharged in cyan and negatively-charged in red. A clear separation between the two regions can be seen (**d**) The surface of the centroid UyCt1 is shown along the *C*-terminal side, with hydrophilic areas in cyan and hydrophobic regions in amber.

Cluster 2, which contains 20 peptides, was also investigated. Its centroid is peptide UyCT1 [37], whose sequence is GFWGKLWEGVKNAI-NH_2_. It is a predominantly hydrophobic peptide, with three aromatic amino acid residues (Phe2, Trp3 and Trp7) and two positively-charged residues (Lys5 and Lys11). It assumes an α-helical conformation after PEP-FOLD prediction, which is maintained during MD simulation (Figure 10b). The same can be said for the majority of the peptides forming cluster 2 (Figure 10a). The disposition of the residues was also analysed and a clear separation between hydrophobic amino acids and positively-charged residues can be observed once again (Figure 10c), in accordance with what previously observed for cluster 1 (Figure 9c). This is mirrored by the analysis of the surface of the centroid UyCT1. A clear distinction between hydrophobic and hydrophilic areas can be seen (Figure 10d), once again confirming what observed for the first set of antimicrobial peptides with experimentally-derived conformations.

The centroids of less populated clusters have less ordered structures (Figure S10), but in most cases form surfaces with amphipathic nature, those sequences can be also used as a starting point for a design of molecules with novel architectures, but the detailed analysis of structures are beyond this work.

### 4.4. MD Simulation to Evaluate Interaction of Short Antimicrobial Peptides with a Membrane-Like Structure

The use of MD simulations for the study of the behaviour of these cationic antimicrobial peptides in the presence of membrane-like structures was evaluated. With respect to this, many NMR experiments use solutions of the peptide in the presence of micelles of amphipathic molecules, whose role is to mimic the membrane. The PDB entry 2MJT, which corresponds to the NMR-derived conformation of the antimicrobial peptide Anoplin in the presence of dodecylphosphocholine (DPC) micelles, was selected as a model system to validate our molecular dynamics simulations setup [11].

**Figure 11:**
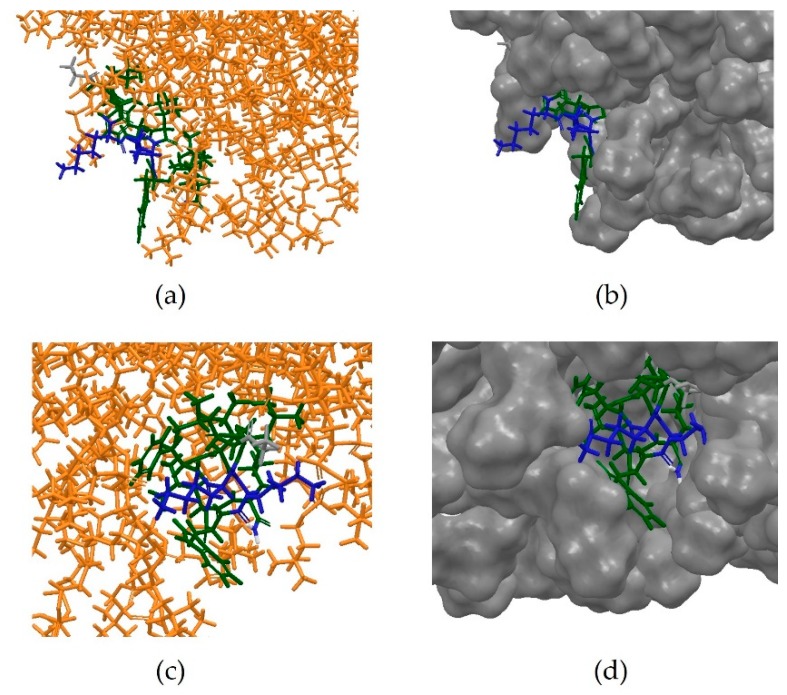
(**a**) Initial frame of the MD simulation of the PDB entry 2MJT; (**b**) Anoplin embedded in the micelle at the beginning of the MD simulations; (**c**) Final frame of the MD simulations; (**d**) Anoplin remains embedded into the micelle at the end of the simulation, showing that this system is stable under the conditions used. The residues have been coloured according to their properties, with positively-charged amino acids in blue, hydrophobic in green, neutral in grey and polar uncharged in cyan. It can be observed that Anoplin maintains an amphipathic conformation throughout the simulation.

Anoplin, a 12-residue *C*-terminally amidated peptide, has been shown to exert its antimicrobial activity through ion channel-like activity in a membrane-like environment [38]. It was shown that this system remains stable after 10 ns MD simulation, as the micelle does not disassemble and Anoplin remains embedded onto its surface, maintaining the characteristic amphipathic structure (Figure 11a–d).

**Figure 12:**
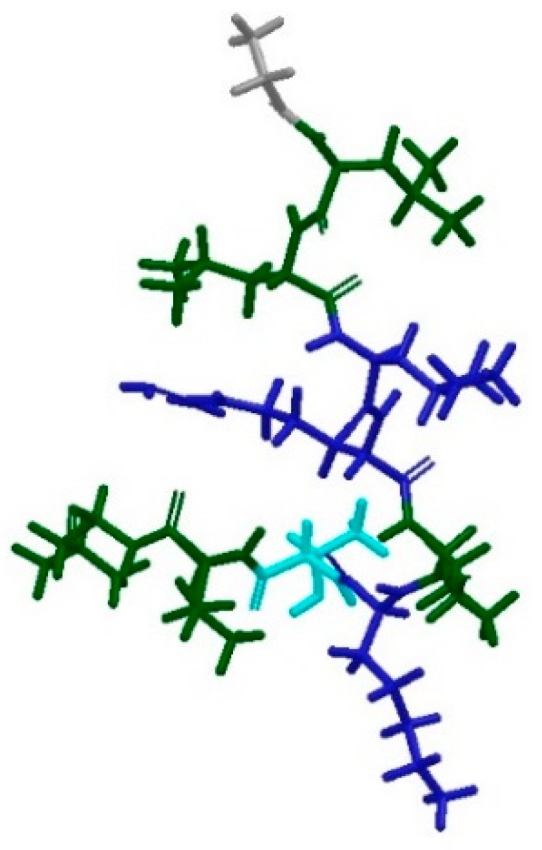
Conformation of extended Anoplin at the end of 10 ns MD simulation. The residues are coloured according to their properties, with electrostatically positively-charged amino acids in blue, hydrophobic in green, polar uncharged in cyan and neutral in grey. It can be observed that the peptide does not assume an amphipathic conformation as expected.

The next step was to design an *in silico* system reproducing the same conditions in which the NMR experiment had been conducted [11]. The same MD protocol was therefore applied to an artificially built system, containing the micelle, the extended peptide built from scratch and the phosphate buffer. However, no interaction between the peptide and the micelle was observed at the end of the simulation, nor did the peptide seem to be able to adopt an amphipathic conformation (Figure 12).

**Figure 13:**
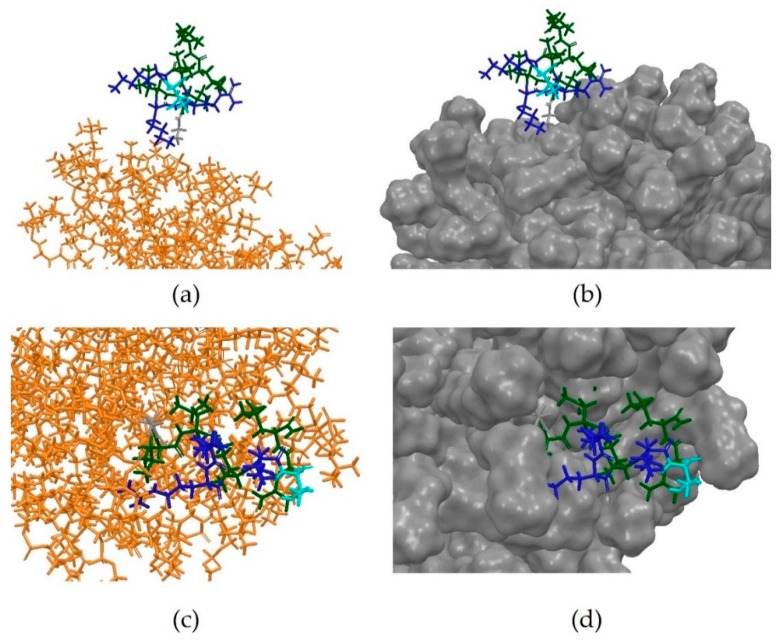
(**a**) Initial frame of the MD simulation on a system including the PEP-FOLD predicted 3D structure of Anoplin; (**b**) Initial frame of the simulation, showing the PEP-FOLD predicted conformation of Anoplin positioned 10 Å away from the surface of the micelle; (**c**) Final frame of the simulation; (**d**) Final frame of the simulation, showing Anoplin embedded into the surface of the micelle in an amphipathic conformation. Residues are coloured according to the surface, with positively-charged amino acids in blue, hydrophobic in green, polar uncharged in cyan and neutral in grey.

The simulation was then repeated under the same conditions but using the PEP-FOLD predicted 3D conformation of Anoplin. This time, a clear interaction between micelle and the peptide was observed, with the latter clearly embedding into the membrane-like surface after assuming an amphipathic conformation (Figure 13a–d).

This is consistent with the previously-observed inability of MD simulations to predict the correct fold from an extended structure even at time-costly simulations of 2 μs. Those long simulations (CPU time) of even relatively small systems comprising peptide, micelle and water molecules are impractical for most academic and industrial groups mainly due to duration (real time) and available computational resources. Therefore, having a realistic starting structure can reduce the need to predict folding of the peptide and significantly decrease the duration of simulations. Here, we have found that PEP-FOLD can predict the fold of AMP that can occur in membrane-like environments, but other methods, not tested here, can be also validated for this use, i.e., PEPstrMOD [39], simulated tempering [40], multiple simulated annealing-molecular dynamics [41] or FlexPepDock [42]. Once the methods are validated, studies can be extended to molecular dynamics simulations of a peptide in the presence of lipopolysaccharide (LPS) micelles, which could provide a more relevant representation of the interactions between the peptide and a surface of a Gram negative bacterium [43].

## 5. Conclusions

The structures and conformations of two sets of cationic antimicrobial peptides were clustered and compared. Importantly, the predicted conformations of AMP were in a good agreement with those determined experimentally, although the presence of a membrane was not explicitly taken into consideration. Consequently, the peptides acting against Gram negative bacteria had their conformations predicted through the online software PEP-FOLD and then submitted to molecular dynamics simulations before being clustered to identify common features possibly responsible for their activity. Significantly, the analysis of the most-populated clusters confirmed that these peptides tend to assume an amphipathic conformation, which is in accordance with their suggested mechanism of action. It was also observed that the majority of these peptides often contain basic amino acids in proximity to aromatic residues. Finally, it was demonstrated that MD simulations in combination with prediction of the initial conformation can be used to study the interaction of short AMPs with a membrane-like structure such as dodecylphosphocholine (DPC) micelles. The results of this study successfully demonstrate that a combination of *in silico* approaches can be utilized to identify common structural features of AMPs and inform the rational design of novel antimicrobial agents.

## Figures and Tables

**Table 1:** Sequences of antimicrobial peptides whose 3D structures were determined in the presence of micelles. For all entries, the NMR data were acquired in the presence of dodecylphosphocholine (DPC) micelles, with the exception of 2MAA which was obtained in presence of lipopolysaccharide (LPS) micelles instead.

PDB ID	Peptide	Sequence	AA
2MJT	Anoplin [11]	GLLKFIKWLL-NH_2_	11
1G89	Indolicidin [12]	ILPWKWPWWPWRR-NH_2_	13
2F3A	LLAA [13]	RLFDKIRQVIRKF-NH_2_	14
1D7N	Mastoparan X [14]	INLKALAALAKKIL-NH_2_	15
2JQ2	PW2 [15]	HPLKQYWWRPST	12
2JMY	CM15 [16]	KWKLFKKIGAVLKVL	15
2MAA	Temporin-1 Ta [17]	FLPLIGRVLSGIL (LPS micelles)	13
1D6X	Tritrpticin [18]	VRRFPWWWPFLRR	13
1T51	IsCT [19]	ILGKIWEGIKSLF-NH_2_	14

**Table 2:** Sequences of antimicrobial peptides whose 3D structure has been determined in the absence of micelles.

PDB ID	Peptide	Sequence	AA
2L24	Not named [20]	IFGAIAGFIKNIW-NH_2_	14
2N9A	Decoralin-NH_2_ [21]	SLLSLIRKLIT-NH_2_	12
2NAL	Retro-KR-12 [22]	RLFDKIRQVIRK	12

**Table 3:** Clusters obtained from hierarchical clustering with threshold set at 700.

Cluster	Number of AMPs in Cluster	RMSD	Centroid PDB ID
1	24	417.506	5MMK
2	14	466.125	2MXQ
3	13	270.961	2NDC
4	13	155.284	2RTV
5	11	183.288	5YKL
6	8	626.605	2LG4
7	7	144.665	2MUH
8	7	3.293	2MLU
9	6	500.236	5UI7
10	5	401.027	2MFS
11	3	333.869	1FRY
12	3	666.667	1ZRX
13	2	1.289	2LS1
14	1	0.000	2N0V

**Table 4:** The root mean square difference (RMSD) values resulting from the superposition of the peptide α-carbon atoms of the *in silico* predicted conformations (PEP-FOLD predicted and extracted from trajectory at 1.2 and 10 ns) on the corresponding experimental determined conformation extracted from the PDB.

PDB Entry	Number of AA	RMSD (Å)		
		PEP-FOLD	1.2 ns	10 ns
2MJT	11	0.4461	0.1793	2.8464
1G89	13	6.4019	6.3364	10.5531
2F3A	14	1.6773	1.4999	1.6041
1D7N	15	2.1988	2.2371	3.1736
2JQ2	12	3.1997	3.5588	3.5946
2JMY	15	1.3061	1.3681	3.0161
2MAA	13	2.0415	2.6618	4.0329
1D6X	13	3.6692	3.3713	4.0437
1T51	14	1.9547	2.4822	2.9325
2L24	14	1.0952	3.0831	5.0254
2N9A	12	1.2719	2.2212	2.6661
2NAL	12	1.3380	1.8398	2.4378

**Table 5:** Clusters obtained from hierarchical clustering with threshold set at 800.

Cluster	Centroid	Size	RMSD
1	VCP-VT1	25	321.152
2	UyCT1	20	550.390
3	Andreonicin C1	3	666.667
4	Tigerin3	2	3.302
5	CP-11C	2	500.00
6	Jelleine II	2	500.00
7	Urechistachykinin I	2	0.320
8	Peptide AN5-1	2	0.323
9	Tigerin 1	1	0.000
10	Cn-AMP1	1	0.000
11	Astadidin 2	1	0.000
12	PGLa-H	1	0.000
13	Odirranain-V1	1	0.000

## Supplementary Materials

The following are available online at http://www.mdpi.com/1999-4923/10/3/72/s1, Table S1: Set of antimicrobial peptides with known 3D structures derived from NMR experiments, Table S2: Set of short sequence antimicrobial peptides, showing their size, sequence, net charge at pH 7.4 and minimum inhibitory concentration (MIC) against some of the most common Gram-negative strains, Figure S1: The experimentally-derived conformations of the PDB entries the centroids of the less populated clusters resulting from hierarchical clustering (HC) of 117 antimicrobial peptides with experimentally-known conformations, Figure S2: Backbone superposition of PDB entry 2F3A (red), with simulated conformations at 0, 1.2 and 10 ns (blue). The conformation at 1.2 ns is very similar to the PDB entry and it progressively modifies during the simulation., Figure S3: Backbone superposition of PDB entry 1D7N (red) with simulated conformations at 0, 1.2 and 10 ns (blue). Superposition slightly decreases over simulation time, suggesting a flexible peptide, especially at the extremities, Figure S4: Backbone superposition of PDB entry 2JMY (red) with simulated conformations at 0, 1.2 and 10 ns (blue). RMS at 0 and 1.2 are quite similar, but similarity decreases over simulation time, suggesting flexibility, especially at the N terminus, Figure S5: Backbone superposition of PDB entry 2MAA (red) and simulated conformations at 0, 1.2 and 10 ns (blue). RMS progressively increases during simulation time, Figure S6: Backbone superposition of PDB entry 1T51 (red) and simulated conformations at 0, 1.2 and 10 ns (blue). Similarity decreases over simulation time, with a distinct flexibility observed at the C terminus, Figure S7: Backbone superposition of PDB entry 2L24 (red) and simulated conformations at 0, 1.2 and 10 ns (blue). As opposed to the peptides analyzed previously, the NMR experiments of 2L24 were acquired in the absence of micelles. However, similarly to the previous cases, similarity also decreases over simulation time, Figure S8: Backbone superposition of PDB entry 2N9A (red) and simulated conformations at 0, 1.2 and 10 ns (blue). 2N9A NMR data were obtained in a simple solution without micelles. Similarity decreases over simulation time, Figure S9: Backbone superposition of PDB entry 2NAL (red) and simulated conformations at 0, 1.2 and 10 ns (blue). NMR data were obtained in the absence of micelles and similarity decreases over simulation time, Figure S10: The conformations centroids of the less populated clusters after hierarchical clustering of the predicted structures of cationic peptides with activity against Gram negative bacteria. References [11,12,14,15,16,18,19,20,21,22,31,33,34,37,44,45,46,47,48,49,50,51,52,53,54,55,56,57,58,59,60,61,62,63,64,65,66,67,68,69,70,71,72,73,74,75,76,77,78,79,80,81,82,83,84,85,86,87,88,89,90,91,92,93,94,95,96,97,98,99,100,101,102,103,104,105,106,107,108,109,110,111,112,113,114,115,116,117,118,119,120,121,122,123,124,125,126,127,128,129,130,131,132,133,134,135,136,137,138,139,140,141,142,143,144,145,146,147,148,149,150,151,152,153,154,155,156,157,158,159,160,161,162,163,164,165,166,167,168,169,170,171,172,173] are cited in the supplementary materials
