# Supplementary material for: In Silico Structural Evaluation of Short Cationic Antimicrobial Peptides
Source: Pharmaceutics. 2018 Jun 21;10(3):72. doi: 10.3390/pharmaceutics10030072 (PMC6160961; doi:10.3390/pharmaceutics10030072)
Supplement: Supplementary file 1 [file pharmaceutics-10-00072-s001.pdf]

pharmaceutics

Supplementary material for "*In silico* structural evaluation of short cationic antimicrobial peptides"

Ilaria Passarini, Sharon Rossiter, John Malkinson, Mire Zloh

Table S1. Set of antimicrobial peptides with known 3D structures derived from NMR experiments.

PDB ID	Peptide	AA
2L24	Not named [20]	14
2L3I	Oxki4A [44]	30
2N8D	Lavracin [45]	21
2NC7	Protegrin PG-5 [46]	18
5YKK	Andersonin-Y1 [47]	18
2GDL	Fowlicidin 2 [48]	31
2HFR	Fowlicidin 3 [49]	27
2KUS	Sm-AMP-1.1a [50]	35
2LG4	Aurelin [51]	40
2LXZ	Defensin 5 [52]	35
2M2G	[Aba3,16]BTD-2 [53]	18
2M2H	[Aba3,7,12,16]BTD-2 [53]	18
2M2S	[Aba5,7,12,14]BTD-2 [53]	18
2M2X	[Aba3,5,7,12,14,16]BTD-2 [53]	18
2M2Y	Btd-2[3-4] [53]	18
2MJT	Anoplin [11]	11
2MLU	LsbB (DPC) [54]	30
2MLV	LsbB (TFE/water) [54]	30
5KI0	KAMP-19 [55]	19
1KFP	Gomesin [56]	18
2RLH	Rp-1 [57]	18
2PCO	Latarcin-1 [58]	26
2N1S	SmAMP2-2c [50]	30
2N1C	PvHCt [59]	23
2MXQ	DEFA1 [33]	34
2MFS	Ep-AMP1 [60]	35
2M6A	Tk-Amp-X2 [61]	28
2L5R	Alyteserin-1C [62]	23
2L1Q	HLE Amp 2 [63]	40
2KNJ	Micropulsin [64]	90
2JNI	Arenicin-2 [65]	21
2AMN	Fowlicidin-1 [66]	36
1MM0	Termicin [67]	36

Table S1. *Cont.*

PDB ID	Peptide	AA
1G89	Indolicidin [12]	14
1FRY	SAMP-29 [68]	29
1CIX	Tachystatin [69]	44
5M9U	Arenicin-1 mutant V8R [70]	21
5YKL	AY1C [71]	19
2LMF	LL-23 [72]	23
1D6X	Tritrpticin [18]	13
1OG7	Sakacin P. [73]	43
1RY3	Carnobateriocin B2 precursor [74]	64
1T51	IsCT [19]	14
2JSB	Arenicin-1 [75]	21
2JOS	Piscidin [76]	22
5X3L	P1 [77]	20
2L2R	EcAMP1 [78]	37
2K98	MSI-594 [79]	24
2G9P	Lataricin 2a [80]	26
2G9L	Gaegurin 4 [81]	37
2DD6	K4-S4(1-13)a [82]	14
2DCX	K4S4(1-13)a [82]	14
2DCW	Tachystatin b [83]	42
2NDC	Bmap-28(1-18) [84]	18
2FBS	LL-37 [85]	14
2MMM	GK cecropin-like [86]	36
1HU5	Ovispirin-1 [87]	18
1HU6	G10 Novispirin [87]	18
1HU7	T7 Novispirin [87]	18
1RKK	Polyphemusin [88]	19
1D7N	Mastoparan X [14]	15
1UEO	[T8A]-Panaeidin-3 [89]	63
1YTR	Plantaricin [90]	26
1Z64	Pleurodicin [91]	26
2A2B	Curvacin A[92]	41
2N92	Cecropin P1 [93]	31
1M4F	Hepcidin-25 [94]	25
1M4E	Hepcidin-20 [94]	
5LAH	Tau-AnmTx Ueq 12-1 [95]	45
2JQ2	PW2 [15]	12
2LTI	Astexin1 [96]	23
2N63	C4VG16KRKP [97]	17
1DQC	Tachycitin [98]	74
2MBD	Lasiocepsin [99]	27
2KEF	Hepcidin [100]	25
2MAA	Temporin-1 Ta [101]	13
2KET	BMAP-27 [102]	27
2NDE	Mutant of BMAP-28(1-18) [103]	18
1X7K	PV5/water [104]	19
2B5K	PV5/DPC [104]	19
1MA2	Tachyplesin I [105]	17
2L9X	Thuricin CD [106]	30

Table S1. *Cont.*

PDB ID	Peptide	AA
5LM0	Tk-hefu [107]	28
5UI6	Acinetodin [108]	18
5UI7	Klebsidin [108]	19
2LS1	Sviceucin [109]	20
2JPK	Lactococcin G-b [110]	35
1CZ6	Androctonin [111]	25
1LFC	LFCINB [112]	25
2RTV	Tachyplesin I [105]	18
2NAU	KYE28A [113]	28
2NAL	Retro-KR-12 [22]	12
2N9A	Decoralin-NH2 [21]	12
2N30	Ace-pvhct-NH2 [59]	25
2MZ6	Protegrin-3 [114]	36
2F3A	LLAA [82]	14
2MUH	Protegrin-2 [115]	16
1PG1	Protegrin-1 [116]	19
2MDB	Tachyplesin I [34]	18
2M60	Enterocin 7B [117]	43
2M5Z	Enterocin 7A [117]	44
2KNS	Pardaxin [118]	33
1ZRX	Stomoxyin [119]	42
5H2S	Tialpia Piscidin 4 [120]	25
5MMK	HYL-20 [31]	16
2K35	Hydramacin-1 [121]	60
1ZFU	Plectasin [122]	40
2KHF	Plantaricin JK [123]	25
2GMC	C12-LF11 [124]	12
2JQ0	Phylloseptin-1 [125]	20
2JPY	Phylloseptin-2 [125]	20
2L5M	GF-17 [126]	18
1MMC	AC-AMP2 [127]	30
1F0D	Cecropin A(1-8)-magainin 2(1-12) [128]	21
2LA2	Papiliocin [129]	38
2JPJ	Lactococcin G-a [110]	39
2N0V	Cn-APM1 [130]	10
2MN5	Copsin [131]	57
2JMY	CM15 [16]	15
2MWT	Crotalicidin [132]	34

Table S2. Set of short sequence antimicrobial peptides, showing their size, sequence, net charge at pH 7.4 and minimum inhibitory concentration (MIC) against some of the most common Gram-negative strains.

Name	Amino Acid Sequence	Net charge	Strain	MIC (µg/mL)
**Jelleine-II** [133]	TPFKISIHL-NH2	2	*E. coli* CCT 1371	15
			K. pneumoniae ATCC 13883	15
			P. aeruginosa ATCC 27853	15
			E. Cloacae ATCC 23355	15
**Jelleine-III** [133]	EPFKISIHL-NH2	2	*E. coli* CCT 1371	15
			P. aeruginosa ATCC 27853	30
**Cn-AMP1** [134]	SVAGRAQGM	1	*E. coli* (not specified)	82
			P. aeruginosa (not specified)	79
			E. coli ATCC 43895	11.97
**Urechistachykinin I** [135]	LRQSQFVGSR-NH2	3	*P. aeruginosa* ATCC 27853	24.06
			V. vulnificus ATCC 29307	48.02
			E. coli ATCC 43895	11.96
**Urechistachykinin II** [135]	AAGMGFFGAR-NH2	2	*P. aeruginosa* ATCC 27853	11.96
			V. vulnificus ATCC 29307	23.92
**PGLa-H** [136]	KIAKVALKAL	4	*E. coli* ATCC 25922	23.6
			E. coli ATCC 25922	30
**Temporin-ALk** [137]	FFPIVGKLLS-NH2	2	*P. aeruginosa* (not specified)	25
			S. dysenteriae (not specified)	60
			Calcoaceticus (not specified)	75
**Balteatide** [138]	LRPAILVRIK	3	*E. coli* NCTC 10418	128
			E. coli ATCC 25922	6.3
			K. pneumoniae ATCC 10031	3.1
**L6K4W6** [139]	KKLLKWLLKLL	4	*P. aeruginosa* ATCC 27853	25
			S. Typhimurium ATCC 14028	25
			S. dysenteriae ATCC 9752	3.1
			E. coli W 160.37	40
**Tigerin 1** [140]	FCTMIPIPRCY-NH2	1	*P. putida* NCIM 2102	40
			Faecalis ATCC 8750	46.67

Table S2. *Cont.*

Name	Amino Acid Sequence	Net charge	Strain	MIC (μg/mL)
**VCP-VT2** [141]	FLPIIGKLLSG	1	*E. coli* ATCC 25922	5
			K. pneumoniae ATCC 700603	10
			P. aeruginosa ATCC 27853	40
			S. Dysenteriae (not specified)	5
			E. cloacae ATCC 13047	2.5
			Pyocyaneus CMCCB 1010	20
**Tigerin 2** [140]	RVCFAIPLPICH-NH2	1	*E. coli* W 160.37	50
			P. putida NCIM 2102	50
**Tigerin 3** [140]	RVCYAIPLPICY-NH2	1	*E. coli* W 160.37	40
			P. putida NCIM 2102	40
**Protonectin** [142]	ILGTILGLLKGL-NH2	2	*E. coli* ATCC 25922	25
			P. aeruginosa ATCC 15442	1.7
**Odorranain-V1** [143]	GLLSGTSVRGSI	1	*E. coli* (not specified)	36
**Myxinidin** [144]	GIHDILKYGKPS	1	*E. coli* D 31	2.0
			P. aeruginosa Z 61	7.0
			P. aeruginosa K 799	10
			S. typhimurium C 610	2.5
			L. anguillarum 02-11	1.0
			Salmonicida A449	2.0
			Y. ruckeri 96-4	2.0
**Temporin-Rb** [145]	FLPVLAGVLSRA	1	*E. coli* HP101BA760C	34.5
			K. pneumoniae PTCC 1388	35.1
			S. Typhimurium PTCC 1428	31.8
**Halictine-2** [146]	GKWMSLLKHILK-NH2	4	*E. coli* (not specified)	8.1
			P. aeruginosa (not specified)	1
**Peptide AN5-1** [147]	YSKSLPLSVLNP	1	*E. coli* ATCC 25922	8
			S. enetriditis ATCC 13076	4
			S. marcescens (not specified)	32
			Enterobacter spp	16

Table S2. *Cont.*

Name	Amino Acid Sequence	Net charge	Strain	MIC (μg/mL)
**VCP-VT1** [141]	FLPIIGKLLSGLL	1	*E. coli* ATCC 25922	2.5
			K. pneumoniae ATCC 700603	10
			P. aeruginosa ATCC 27853	40
			S. dysenteriae (not specified)	20
			E. cloacae ATCC 13047	2.5
			Pyocyaneus CMBCCB 1010	10
**Mastoparan-VT2** [141]	NLKAIAALAKKLL	3	*E. coli* ATCC 25922	20
			K. pneumoniae ATCC 700603	20
			P. aeruginosa ATCC 27853	40
			S. dysenteriae (not specified)	20
			E. cloacae ATCC 13047	10
			B. pyocyaneus CMBCCB 1010	20
**Odorranain-G1** [143]	FMPILSCSRFKRC	3	*E. coli* (not specified)	4.68
**Temporin A** [148]	FLPLIGRVLSGIL-NH2	2	*E. coli* D21	15.49
			Y. pseudotuberculosis (not specified)	26.04
**Crabrolin-3A** [149]	FLALILRKIVTAL-NH2	2	*E. coli* W 160.37	60
**IndolicidinC** [150]	ILPWKWPWWPWRR-CH3	4	*E. coli* UB 1005 wild type	16
			E. coli D2 antibiotic super susceptible strain	4
			K. pneumoniae H103 wild type	64
			K. pneumoniae K799 wild type	64
			K. pneumoniae Z61 antibiotic super susceptible strain	4
			S. typhimurium 14028S wild type	64
			S. typhimurium MS7953S (defensin super susceptible strain)	8

Table S2. *Cont.*

Name	Amino Acid Sequence	Net charge	Strain	MIC (µg/mL)
**CP-11C** [150]	ILKKWPWWPWRRK-CH3	6	*E. coli* UB1005 (wild type)	2
			E. coli DC2 (antibiotic super susceptible strain)	2
			K. pneuminiae H103 (wild type)	8
			K. pneumoniae K799	8
			Z61 (antibiotic super susceptible strain)	2
			S. typhimurium 14028s (wild type)	
			S. typhimurium MS7953s (defensin-super susceptible strain)	1
**Temporin-1Oa** [151]	FLPLLASLFSRLL-NH2	2	*E. coli* ATCC 25922	97.72
**Temporin -1Va** [152]	FLSSIGKILGNLL-NH2	2	*E. coli* ATCC 25922	51.52
			E. cloacae HNTCC 53001	51.52
**VESP-VB1** [153]	FMPIIGRLMSGSL	1	*E. coli* ATCC 25922	7.5
			E. coli 23A (clinical isolate drug resistant strain against ampicillin chephalothin I, II, III, IV)	7.5
			E. coli 27A (clinical isolate drug resistant strain against ampicillin, chephalothin I, II, III, IV)	15
			P. aeruginosa 3A (clinical isolated multidrug resistant strain against methicillin amoxicillin ampicillin cephalothin I II III IV)	3.75

Table S2. *Cont.*

Name	Amino Acid Sequence	Net charge	Strain	MIC (μg/mL)
**UyCT2** [37]	FWGKLWEGVKNAI-NH2	2	*E. coli* ATCC 25922	36.88
			P. aeruginosa ATCC 9027	59
**UyCT5** [37]	IWSAIWSGIKGLL-NH2	2	*E. coli* ATCC 25922	20.48
			P. aeruginosa ATCC 9027	2.73
**Pantinin3** [154]	FLSTIWNGIKSLL	1	*E. coli* DH5α	22.55
**Temporin-1Sa** [155]	FLSGIVGMLGKLF-NH2	2	*E. coli* ATCC 25922	13.02
			E. coli ATCC 35218	13.02
			P. aeruginosa ATCC 27853	40.37
**Meucin-13** [156]	IFGAIAGLLKNIF-NH2	2	*E. coli* ATCC 25922	10.23
			S. typhimutium CCTCCAB 94007	>64.75
			A. Tumerfaciens (not specified)	15.28
			S. oneidensis (not specified)	8.03
**Anderonin-C1** [141]	TSRCIFYRRKKCS	5	*E. coli* (not specified)	30
**Temporin-Ali** [137]	FFPIVGKLLSGLL-NH2	2	*E. coli* ATCC 25922	7.5
			P. aeruginosa (not specified)	5
			S. dysentheriae (not specified)	20
			A. calcoaceticus (not specified)	60
**BmKn2** [157]	FIGAIARLLSKIF	3	*E. coli* (not specified)	1.5
			P. aeruginosa (not specified)	21.3
**IsCT2** [158]	IFGAIWNGIKSLF	2	*E. coli* CCT 1371	4.73
			E. coli ATCC 25922	9.46
			P. aeruginosa ATCC 278536	92.58
**VmCT1** [159]	FLGALWNVAKSVF-NH2	1	*E. coli* ATCC 25922	34.50
			P. aeruginosa ATCC 9027	13.80
			S. typhi ATCC 6399	6.9
**VmCT2** [159]	FLSTLWNAAKSIF-NH2	1	*E. coli* ATCC 25922	28.52
			P. aeruginosa ATCC 9027	14.26
			S. typhi ATCC 6399	14.26
**Temproin-1KM** [160]	FIPLVSGLFSRLL-NH2	2	*E. coli* ATCC 25922	12.5

Table S2. *Cont.*

Name	Amino Acid Sequence	Net charge	Strain	MIC (μg/mL)
**Pleurain B1** [161]	FLGGLIASLLGKI	1	*E. coli* ATCC 25922	25
**Spiniferin-M** [162]	ILGKIWKGIKNIL-NH2	3	*S. enetriditis* AB2010185	44.76
**K4** [163]	KKKKPLFGLFFGLF	4	*E. coli* (not specified)	5.7
**Mastoparan-VT1** [141]	INLKAIAALAKKLL	3	*E. coli* ATCC 25922	20
			K. pneumoniae ATCC 700603	10
			P. aeruginosa ATCC 27853	5
			S. dyssenteriae (not specified)	5
			E. cloacae ATCC 13047	20
			B. pyocyaneus CMCCB 1010	40
**Odorranain-S1** [143]	FLPPSPWKETFRTS	1	*E. coli* (not specified)	42.5
**Japonicin-1CDYa** [164]	FFPLALLCKVFKKC	3	*E. coli* ATCC 11775	39.21
**D-1CDYa** [164]	IIPLPLGYFAKKT	2	*E. coli* ATCC 11775	4.71
**Temporin-1CSd** [165]	NFLGTLVNLAKKIL-NH2	3	*E. coli* ATCC 25726	92.49
**MP-VB1** [166]	INMKASAAVAKKLL	3	*E. coli* ATCC 25922	15
			E. coli 23A (clinical isolate)	15
			E. coli 27A (clinical isolate)	60
			P. aeruginosa 3A (clinical isolate)	15
**Eumenene-mastoparan-AF** [167]	INLLKIAKGIIKSL-NH2	3	*E. coli* CCT1371	20
			E. coli ATCC 25922	50
			E. coli ATCC 25922	25
			P. aeruginosa ATCC 15442	20
**Pd_mastoparan PDD-A** [168]	INWKKIFEKVKNLV-NH2	4	*E. coli* (not specified)	12.43
**Mp_mastoparan MP** [168]	INWLKLGKKMMSAL-NH2	4	*E. coli* (not specified)	99.79
**Polybia-MP-I** [169]	IDWKKLLDAAKQIL-NH2	2	*E. coli* CCT 1457	8
			P. aeruginosa ATCC 15442	8
**Astacidin 2** [170]	RPRPNYRPRPIYRP-NH2	6	*E. coli* D 21	3.9
			P. aeruginosa OT97	7.8
			P. vulgaris OX19 ATCC 6380	7.8
			S. flexneri ATCC 203	0.98

Table S2. *Cont.*

Name	Amino Acid Sequence	Net charge	Strain	MIC (µg/mL)
**Temporin-Ra** [145]	FLKPLFNAALKLLP	2	*E. coli* HP101BA7601c	37.14
			K. pneumoniae PTCC1388	47.90
**UyCT1** [37]	GFWGKLWEGVKNAI-NH2	2	*E. coli* ATCC 25922	16.06
			P. Aeruginosa ATCC 9027	16.0
**Pantinin1** [154]	GILGKLWEGFKSIV	1	*E. coli* DH5α	95
			S. enetriditis AB2010185	110.36
			E. cloacae AB2010162	116.49
**Mastoparan-VT4** [141]	INLKAIAPLAKKLL	3	*E. coli* IS 117#	80
			E. coli IS218#	80
			K. pneumoniae ATCC 700603	20
			P. aeruginosa ATCC 27853	10
			P. aeruginosa IS350#	80
			S. dysenteriae (not specified)	10
			E. cloacae ATCC 13047	40
			B. pyocyaneus CMCCB 1010	10
**Hylain 2** [171]	GILDPIKAFAKAAG-NH2	2	*E. coli* ATCC 25922	22
			P. aeruginosa (not specified)	11
			S: dysentheriae (not specified)	5.5
**Dybowskin-4** [172]	VWPLGLVICKALKIC	2	*E. coli* KCTC 2433	15
			K. pneumoniae ATCC 10031	15
			S. dysenteriae ATCC 9752	30
**Odorranain-T1** [143]	TSRCYIGYRRKVVCS	4	*E. coli* (not specified)	17.5
**Eumenitin** [173]	LNLKGIFKKVASLLT	3	*E. coli* CCT 1371	9.79
			E. coli ATCC 25922	9.79
			P. aeruginosa ATCC 15442	48.99
			E. cloacae ATCC 23355	>97.98
			P. mirabilis CS	>97.98

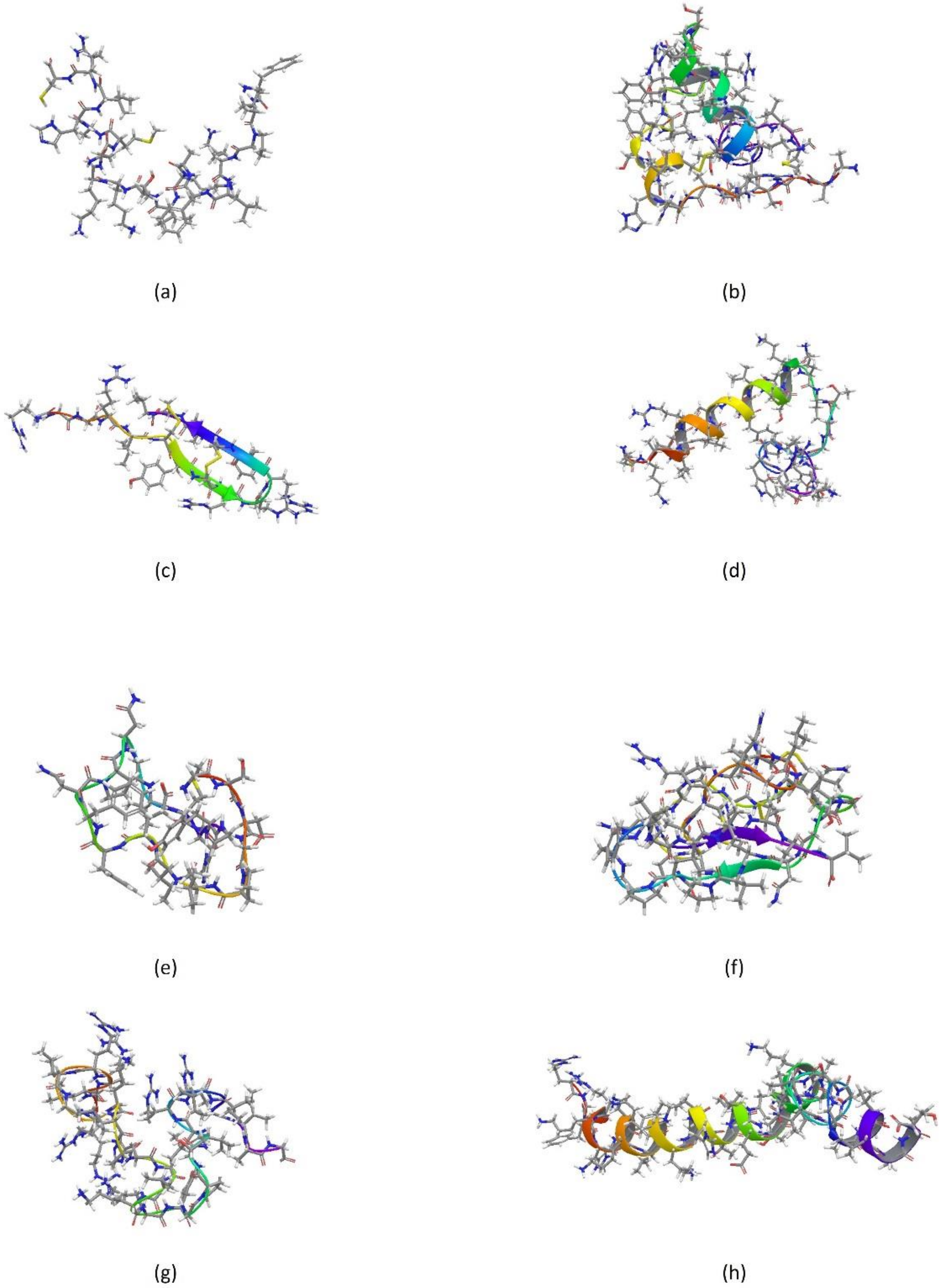

Figure S1 . *Cont.*

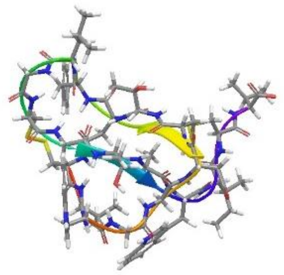

(i)

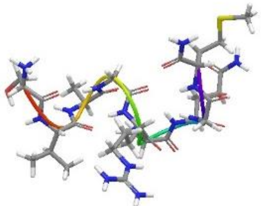

(j)

Figure S1. The experimentally derived conformations of the PDB entries the centroids of the less populated clusters resulting from hierarchical clustering (HC) of 117 antimicrobial peptides with experimentally known conformations.
(a) 5YKL (LPKLFAKITKKNMAHIRC), cluster size = 11;
(b) 2LG4 (AACSDRAHGHICESFKSFCKDSGRNGVKLRANCKKTCGLC), cluster size = 8;
(c) 2MUH (RGGRLCYCRRRFCVCV), cluster size = 7;
(d) 2MLU (MKTILRFVAGYDIASHKKKTGGYPWERGKA), cluster size = 7;
(e) 5UI7 (GSDGPIIEFFNPNGVMHYG), cluster size = 6;
(f) 2MFS (CVLIGQRCDNDRGPRCCSGQGNCVPLPFLGGVCAV), cluster size = 5;
(g) 1FRY (RGLRRLGRKIAHGVKKYGPTVLRIIRIAG), cluster size = 3;
(h) 1ZRX (RGFRKHFNKLVKKVKHTISETAHVAKDTAVIAGSGAAVVAAT), cluster size = 3;
(i) 2LS1 (CVWGGDCTDFLGCGTAWICV), cluster size = 2;
(j) 2N0V (SVAGRAQGM-NH_2), cluster size = 1.

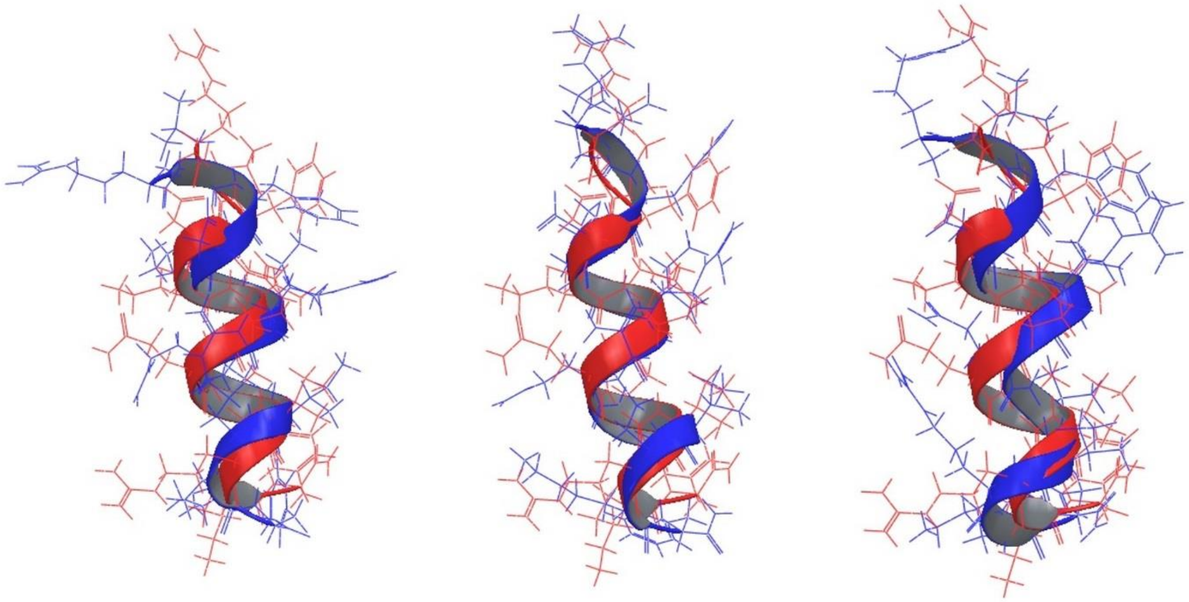

0 ns 1.2 ns 10 ns

Figure S2. Backbone superposition of PDB entry 2F3A (red), with simulated conformations at 0, 1.2 and 10 ns (blue). The conformation at 1.2 ns is very similar to the PDB entry and it progressively modifies during the simulation.

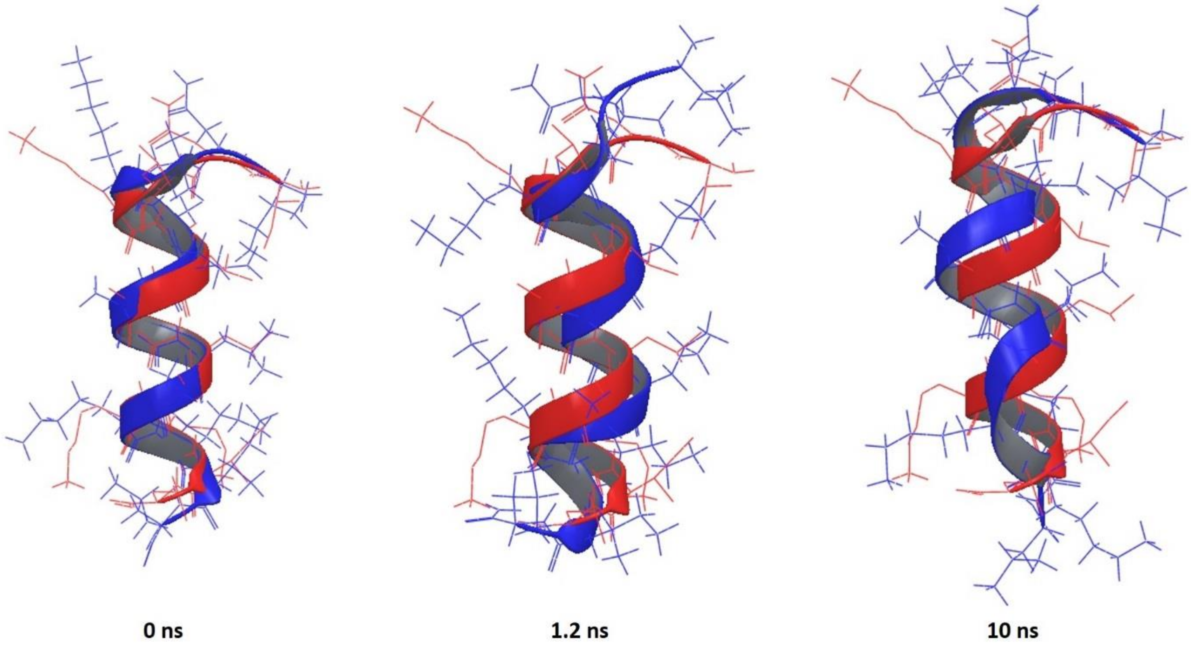

Figure S3. Backbone superposition of PDB entry 1D7N (red) with simulated conformations at 0, 1.2 and 10 ns (blue). Superposition slightly decreases over simulation time, suggesting a flexible peptide, especially at the extremities.

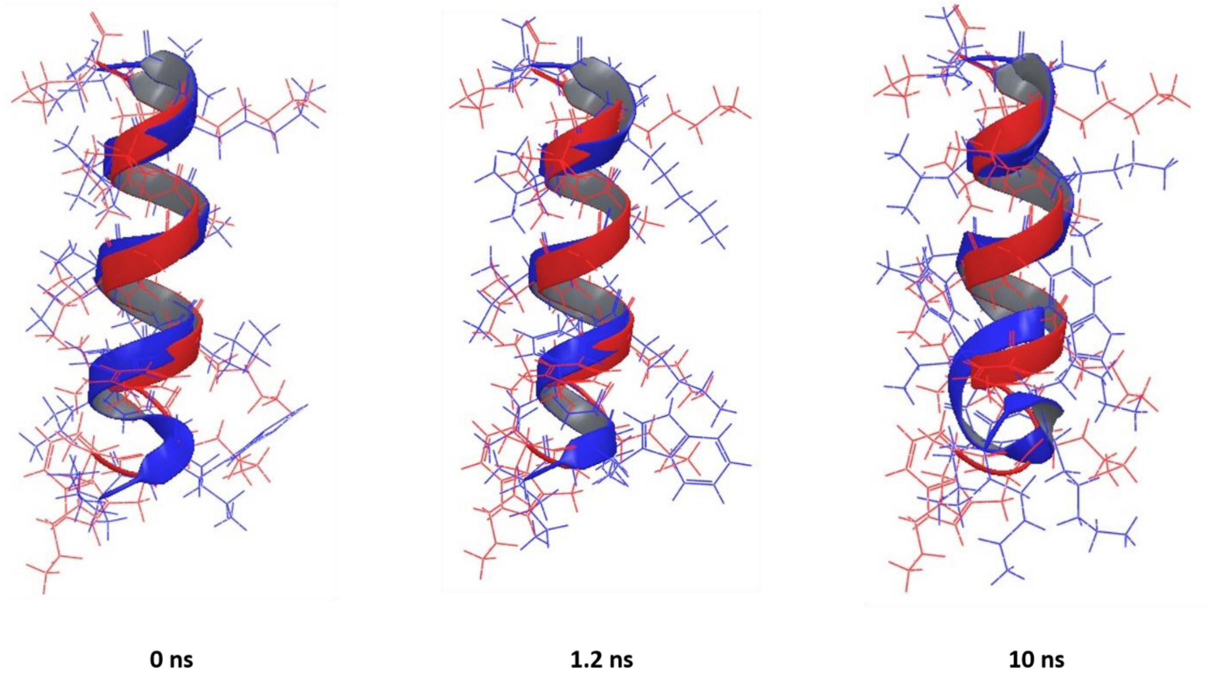

Figure S4. Backbone superposition of PDB entry 2JMY (red) with simulated conformations at 0, 1.2 and 10 ns (blue). RMS at 0 and 1.2 are quite similar, but similarity decreases over simulation time, suggesting flexibility, especially at the N terminus.

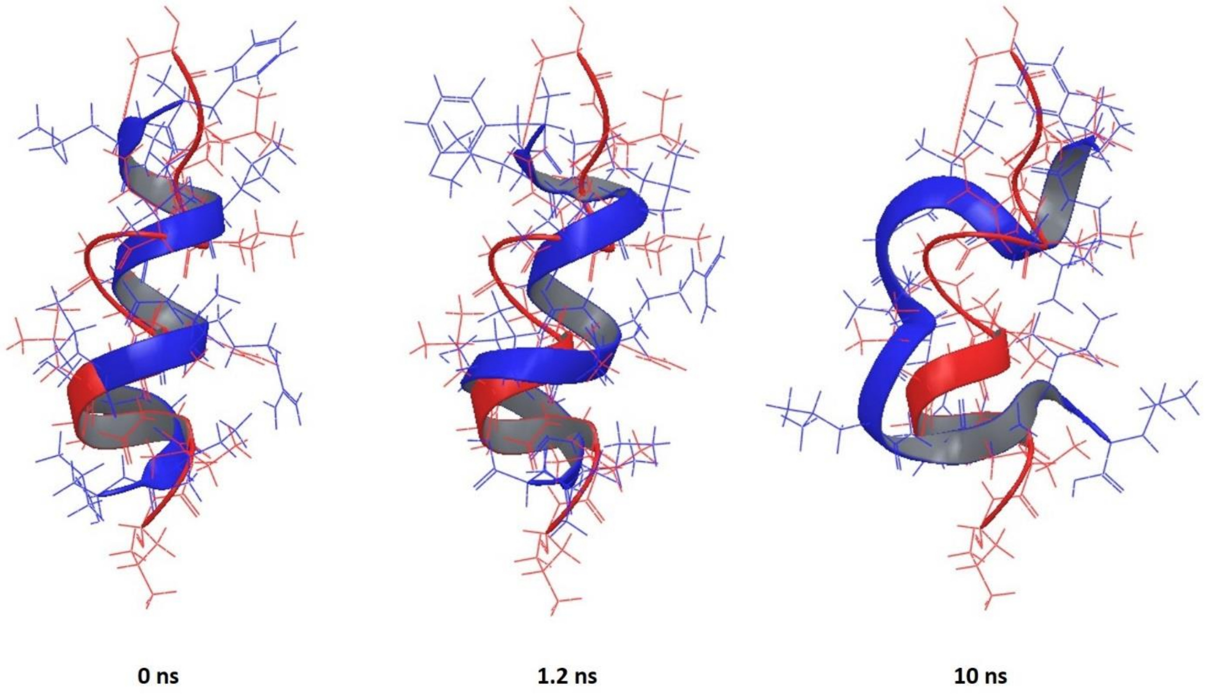

Figure S5. Backbone superposition of PDB entry 2MAA (red) and simulated conformations at 0, 1.2 and 10 ns (blue). RMS progressively increases during simulation time.

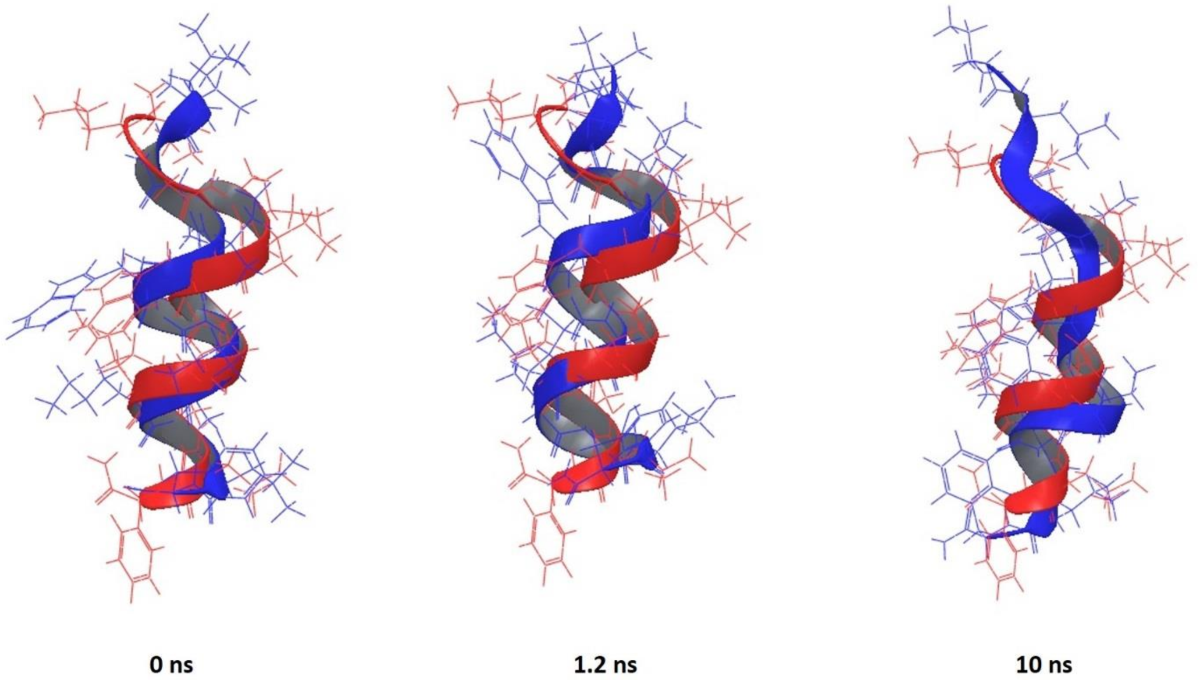

Figure S6. Backbone superposition of PDB entry 1T51 (red) and simulated conformations at 0, 1.2 and 10 ns (blue). Similarity decreases over simulation time, with a distinct flexibility observed at the C terminus.

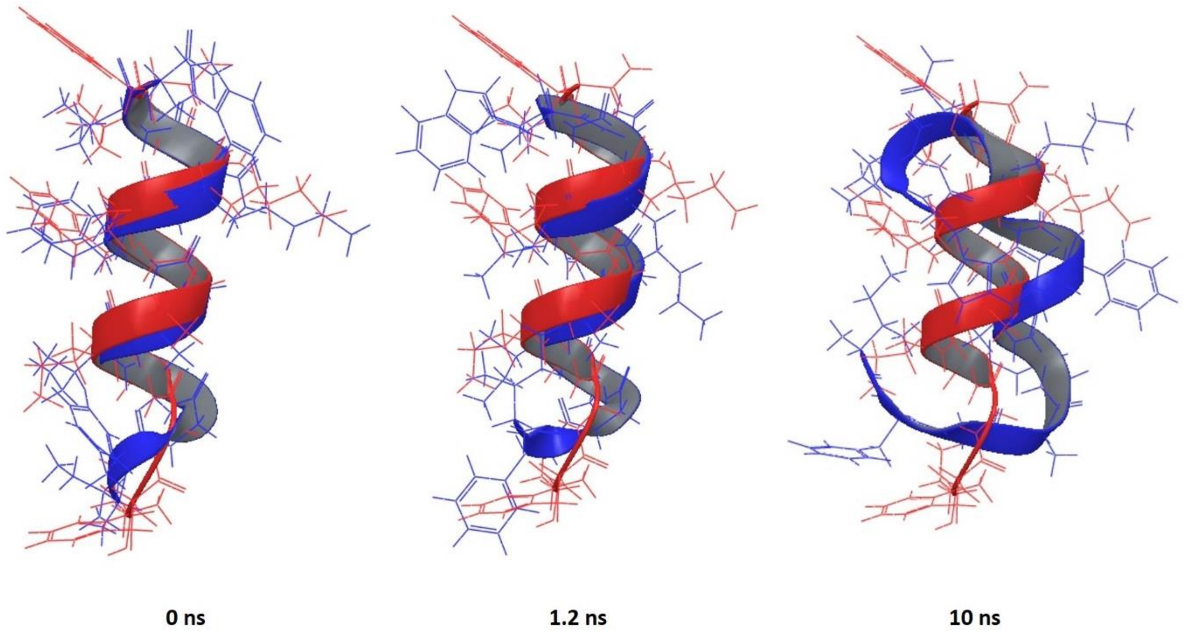

Figure S7. Backbone superposition of PDB entry 2L24 (red) and simulated conformations at 0, 1.2 and 10 ns (blue). As opposed to the peptides analyzed previously, the NMR experiments of 2L24 were acquired in the absence of micelles. However, similarly to the previous cases, similarity also decreases over simulation time.

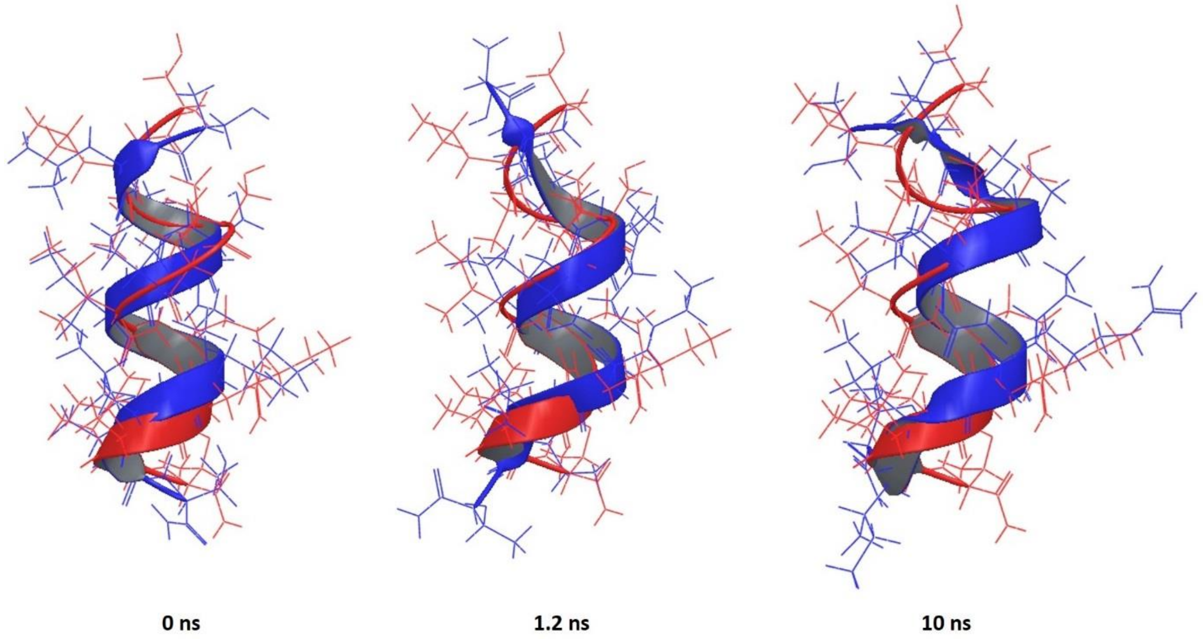

Figure S8. Backbone superposition of PDB entry 2N9A (red) and simulated conformations at 0, 1.2 and 10 ns (blue). 2N9A NMR data were obtained in a simple solution without micelles. Similarity decreases over simulation time.

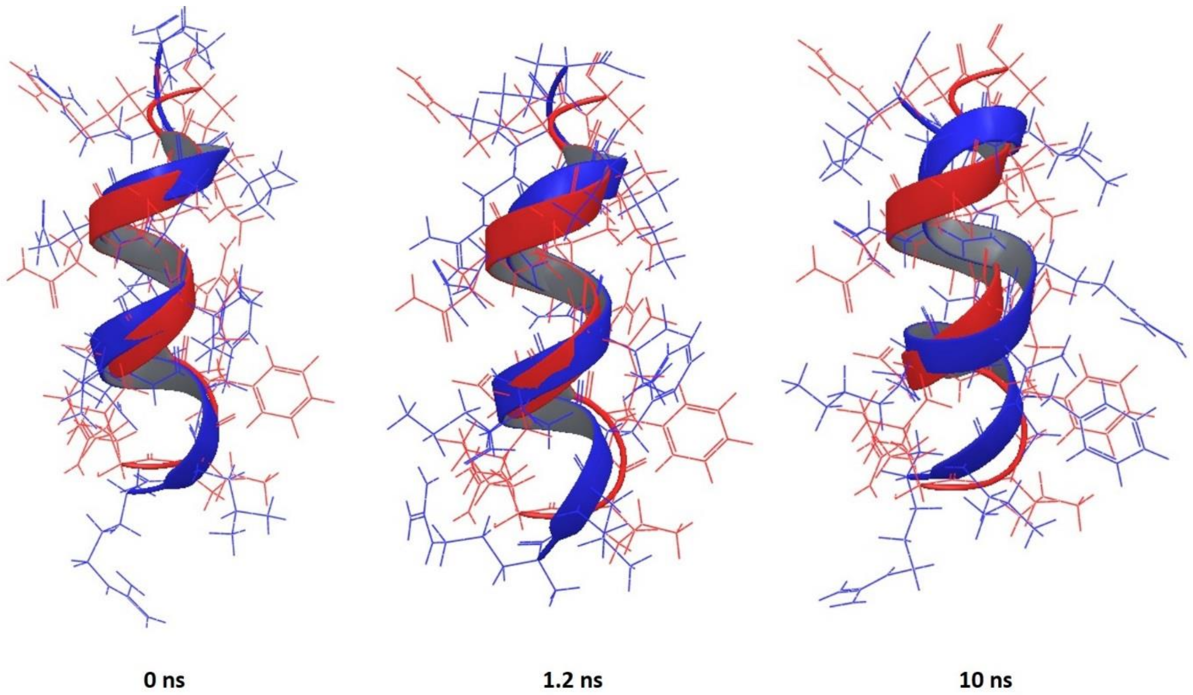

Figure S9. Backbone superposition of PDB entry 2NAL (red) and simulated conformations at 0, 1.2 and 10 ns (blue). NMR data were obtained in the absence of micelles and similarity decreases over simulation time.

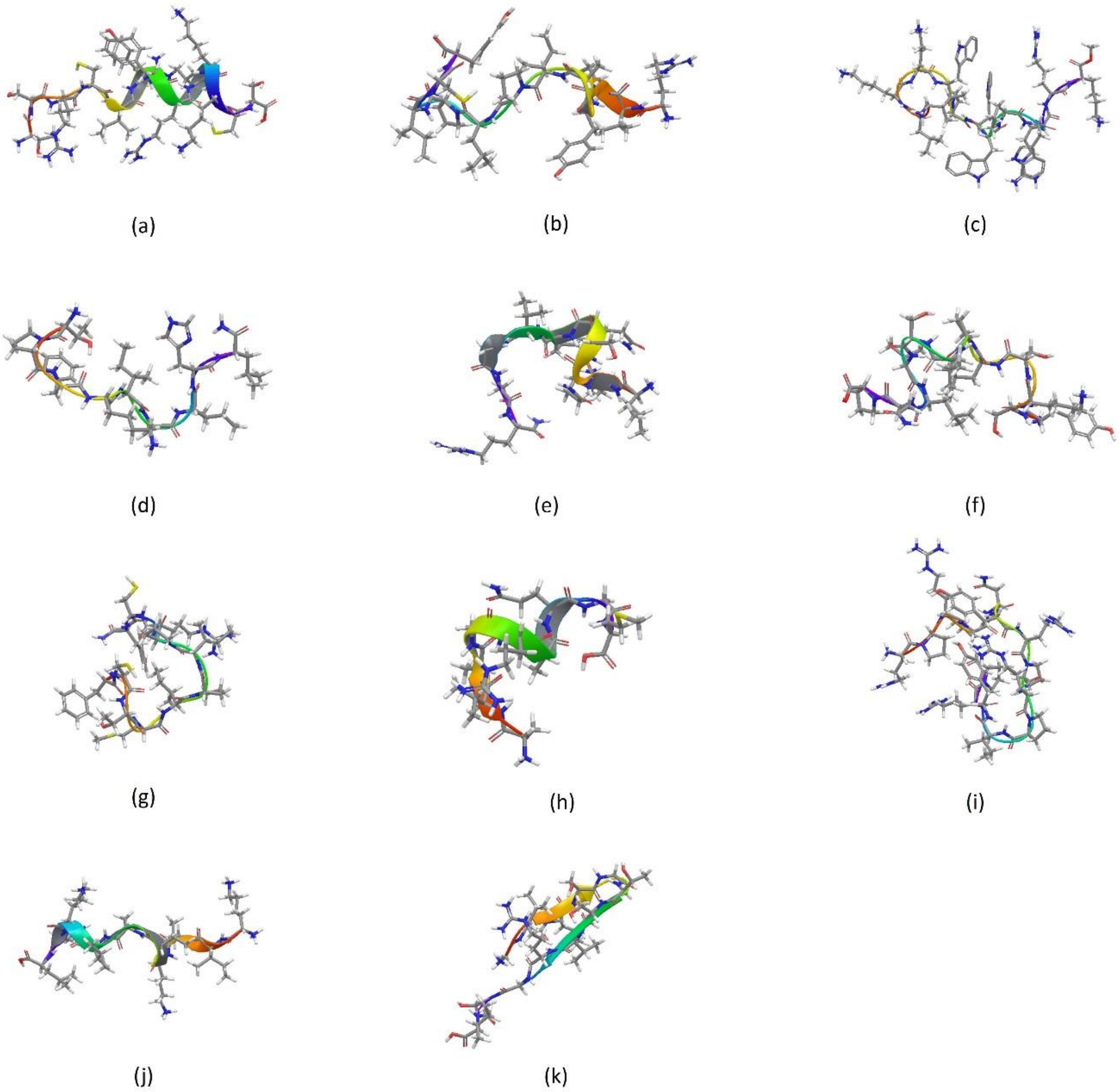

Figure S10. The conformations of centroids of the less populated clusters after hierarchical clustering of the predicted structures of cationic peptides with activity against Gram negative bacteria.
(a) Andreonin-C1 (TSRCIFYRRKKCS), cluster size = 3;
(b) Tigerin 3 (RVCYAIPLPICY), cluster size = 2;
(c) CP-11C (ILKKWPWWPWRRK-CH_3), cluster size = 2;
(d) Jelleine-II (TPFKISIHL-NH_2), cluster size = 2;
(e) Urechistachynin I (LRQSQFVGSR-NH_2), cluster size = 2;
(f) Peptide AN5-1 (YSKSLPLSVLNP), cluster size = 1;
(g) Tigerin 1 (FCTMIPIPRCY-NH_2), cluster size = 1;
(h) Cn-AMP1 (SVAGRAQGM), cluster size = 1;
(i) Astacidin 2 (RPRPNYRPRPRPIYRP-NH_2), cluster size = 1;
(j) PGLaH (KIAKVALKAL) cluster size = 1;
(k) Odorranain-V1 (GLLSGTSVRGSI) cluster size = 1.
